# Supervised Statistical Learning Prediction of Soybean Varieties and Cultivation Sites Using Rapid UPLC-MS Separation, Method Validation, and Targeted Metabolomic Analysis of 31 Phenolic Compounds in the Leaves

**DOI:** 10.3390/metabo11120884

**Published:** 2021-12-17

**Authors:** Chan-Su Rha, Eun Kyu Jang, Yong Deog Hong, Won Seok Park

**Affiliations:** 1 AMOREPACIFIC R&D Center, Yongin 17074, Korea; hydhong@amorepacific.com (Y.D.H.); wspark@amorepacific.com (W.S.P.); 2 Gyeonggi-do Agricultural Research & Extension Services, Hwaseong 18388, Korea; jek0428@gg.go.kr

**Keywords:** chemometrics, flavonoid, machine learning, multivariate analysis, non-conventional edible plants, soybean leaf, targeted metabolomics

## Abstract

Soybean (*Glycine max*; SB) leaf (SL) is an abundant non-conventional edible resource that possesses value-adding bioactive compounds. We predicted the attributes of SB based on the metabolomes of an SL using targeted metabolomics. The SB was planted in two cities, and SLs were regularly obtained from the SB plant. Nine flavonol glycosides were purified from SLs, and a validated simultaneous quantification method was used to establish rapid separation by ultrahigh-performance liquid chromatography-mass detection. Changes in 31 targeted compounds were monitored, and the compounds were discriminated by various supervised machine learning (ML) models. Isoflavones, quercetin derivatives, and flavonol derivatives were discriminators for cultivation days, varieties, and cultivation sites, respectively, using the combined criteria of supervised ML models. The neural model exhibited higher prediction power of the factors with high fitness and low misclassification rates while other models showed lower. We propose that a set of phytochemicals of SL is a useful predictor for discriminating characteristics of edible plants.

## 1. Introduction

**Figure 1:**
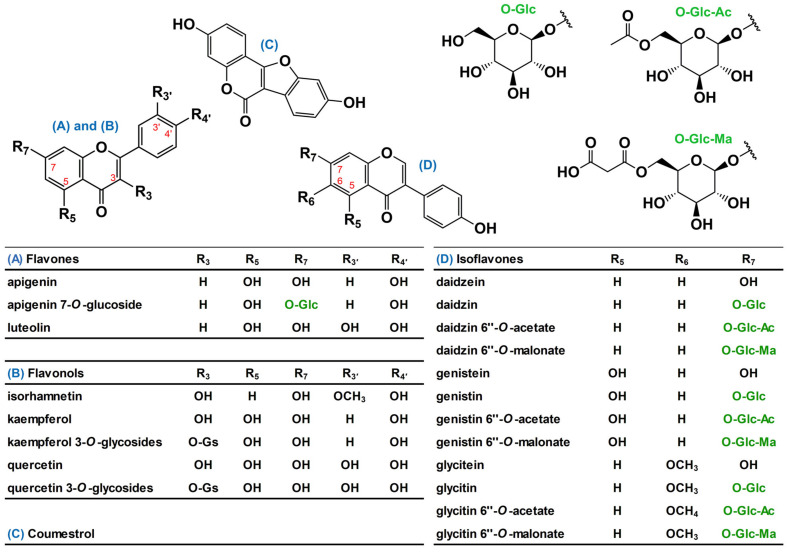
Representative phenolic compounds presented in soybean (SB) leaves. Hereafter, chemical names of three acetate- and three malonate isoflavones were used as acetyl- and malonyl isoflavone, respectively, for convenience. Chemical symbols in the table: *O*-Gs, *O*-glycosides include mono-, di-, tri-glycosides; *O*-Glc, 1-dehydro-glucose; *O*-Glc-Ac, acetyl 1-dehydro-glucose; and *O*-Glc-Ma, malonyl 1-dehydro-glucose.

Soybean (SB; *Glycine max* (L.) Merrill) is one of the most widely cultivated edible plants in the world for human and animal nutrition; it is rich in plant-based proteins and oils [1]. SB plant is a dicotyledonous annual plant that is characterized in two distinct stages; vegetative (V_1_–V_n_) and reproductive (R_1_–R_8_) stages. The appearance of SB seeds is similar among varieties except for coat color, while the chemical compositions of them are different across varieties and environmental factors [2]. Phytochemically, SB seeds primarily contain isoflavones, such as daidzin and genistin, which accumulate during seed maturation via an endogenous metabolic pathway when isoflavone synthase is expressed in SB plant tissues during development [2,3]. In addition, isoflavones, flavonols, flavones, and glycosides are present in soybean leaves (SLs) in the form of aglycones and glycosides of apigenin, kaempferol, and quercetin (Figure 1) [4,5]. Intrinsic and extrinsic factors such as the variety, latitude of the plantation site, fertilization, and climate cause different metabolic responses in SLs because of many polyphenol synthetases in the metabolic pathway [6]; however, there is limited information on the correlation between SB variety and phenolic composition of the SL [7]. Moreover, it is difficult to distinguish mature SB plant characteristics by the appearance of the beans and the leaves in the early stages of the SB plant [2]. Therefore, it is worthwhile to predict or discriminate SB plant characteristics during the cultivation period, including earlier stages, using chemometric information of SLs.

Given the importance of SB as a prominent food source, metabolomic approaches for the leaf have delivered discriminable capacities to an SB plant breeder and developer, such as geographical dependency [8], genotype variability [9], and various environmental responses [10,11,12]. Furthermore, predictive tools are relatively new and highly demanding in the area of plant metabolomics. To date, no universal method is available to identify predictive metabolic responses; accordingly, targeted metabolomics is preferred to establish a predictive model even with a smaller number of markers [12].

Nontargeted metabolomic analysis of food phenolics in plants can provide abundant information using advanced analytical instruments [13,14]. However, it requires prerequisites and conditions, such as quality control/quality assurance (QC/QA) samples and expensive hardware and software. Although over 100 compounds in SB samples have been identified using high-resolution instruments, the content of each compound may not be quantified without authentic standard compounds [15]. Many studies have focused on SB isoflavonoids; however, the targets of these analyses have been limited because conjugated isoflavones were predominant [1,16,17,18]. To investigate targeted metabolomic changes in SB plants during growth, diverse chemicals should be considered [19]. To date, no study has investigated the correlation between the phenolic composition of SL and the phenotype of SB.

It is essential to analyse complex and large data sets of metabolomic subjects; accordingly, various statistical tools have been developed and have continuously improved abilities regarding data analysis. Among many multivariate analyses, principal component analysis (PCA) and orthogonal partial least-squares discriminant analysis (OPLS-DA) have been commonly used in the area of metabolomics. Recently, publications using other potential machine learning (ML) tools, which possess diverse algorithms and performances compared with the aforementioned tools, have substantially increased; however, little literature has been reported for comparative work in the area of plant and agricultural science [20].

Here, we hypothesized that the secondary metabolites of SL can discriminate the difference between the phenotype and cultivation information of SB plants. We prepared nine purified flavonol glycosides from SLs and determined the content of 31 compounds in SL of SB plant varieties using ultrahigh-performance liquid chromatography (UPLC) system coupled with electrospray ionization (ESI) single quadrupole mass spectrometer (MS). A rapid simultaneous separation (<7 min) method validation was established, and various supervised ML models were applied to differentiate the key compounds by cultivation day, planting site, and variety. Further, we compared and determined the prediction power for discriminating these differences by ML models.

## 2. Results and Discussion

### 2.1. Identification of Purified Flavonol Glycosides

We observed distinctive peak profiles for the SLs across varieties. Accordingly, two or three flavonol glycosides were majorly presented with different retention times (RTs) in the separation chromatography for each SL. Nine flavonol glycosides were separated and purified by preparative HPLC, based on the peak collection (Figure S1; Supplementary Materials). The separated peaks were tentatively identified as quercetin or kaempferol glycosides. The flavonol glycosides were identified as quercetin 3-*O*-triglycosides of Cheng-ja no.3 soybean variety (CJ), kaempferol 3-*O*-triglycosides of Dae-won soybean variety (DW), quercetin 3-*O*-diglycosides of Nok-poong soybean variety (NP), and kaempferol 3-*O*-di and triglycosides of Woo-ram soybean variety (WR) (Table 1 and Figures S2–S5). The values of the molecular mass of the precursors and fragments were identical to those of previous studies [4,21,22], and no further structural analysis (nuclear magnetic resonance) was performed. Kaempferol and quercetin glycosides were the dominant phenolics in yellow bean varieties (DW and WR) and colored bean varieties (CJ and NP), respectively. The acceptable molecular structures of the nine flavonol glycoside can be suggested according to the references as follow; CJ1, quercetin 3-*O*-β-d-glucopyranosyl(1⟶2)-*O*-[α-l-rhamnopyranosyl(1⟶6)]-β-d-galactopyranoside; CJ2, quercetin 3-*O*-β-d-glucopyranosyl(1⟶2)-*O*-[α-l-rhamnopyranosyl(1⟶6)]-β-d-glucopyranoside; DW1, kaempferol 3-*O*-β-d-glucopyranosyl(1⟶2)-*O*-[α-l-rhamnopyranosyl(1⟶6)]-β-d-galactopyranoside; DW2, kaempferol-3-*O*-β-d-glucopyranosyl(1⟶2)-*O*-[α-l-rhamnopyranosyl(1⟶6)]-β-d-glucopyranoside; NP1, quercetin 3-*O*-β-d-glucopyranosyl(1⟶2)-β-d-galactopyranoside; NP2, quercetin 3-*O*-β-d-glucopyranosyl(1⟶2)-β-d-glucopyranoside; WR1, kaempferol 3-*O*-α-l-rhamnopyranosyl(1⟶2)-*O*-[α-l-rhamnopyranosyl(1⟶6)]-β-d-galactopyranoside; WR2, kaempferol 3-*O*-α-l-rhamnopyranosyl(1⟶6)-β-d-galactopyranoside; and WR3, kaempferol-3-*O*-α-l-rhamnopyranosyl(1⟶6)-β-d-glucopyranoside.

The spectral purities of the purified flavonol glycosides were calculated in the range of 64–99% by impurity subtraction based on the peak areas in the Max-ultraviolet (UV) chromatogram (210–800 nm) using a photodiode array (PDA) detector (Figures S6–S14). If an impurity in the purified compounds could be separated in a UV chromatogram, the area % of impurity was subtracted from the purity of the compound. If an impurity could not be separated in UV but could be detected using an MS, the quantified values of the impurities were considered. The modified purities for quantification were calculated using a combined solution of 31 compounds (Table S1). It was inevitable to retain certain impurities because of the chemical characteristics, which were difficult to separate using a C_18_ column. A more precise separation will be considered, such as chiral chemistry, in the future. We considered this premise meaningful for executing a targeted metabolomic approach with appropriate chemical diversity of SLs.

### 2.2. Separation Method Validation

The 31 compounds were separated within a run time of 7 min using UPLC-MS and C18 columns. Separation strategies were organized into three parts: hydrophilic (peak nos. **1**–**9**), mesohydrophilic (peak nos. **10**–**21**), and hydrophobic parts (peak nos. **22**–**31**) (Figure S15). Owing to mutual interaction under changing elution conditions, a peak overlap occurred between apigenin (**28**) and genistein (**27**), which have the same molecular weight. Except for these two, all peaks were separated with a proper resolution during mass detection. (Figure S15). Mass detection was conducted within a limited RT window of acquisition time to enhance sensitivity by improving interscan capacity during simultaneous acquisition and yielding a valid number of quantification points for one peak [23,24]. The symmetry factors of the 31 compounds ranged from 0.9 to 1.20 for mass detection (data not shown).

Linearity, limit of detection (LOD), limit of quantification (LOQ), precision, and trueness expressed as a percentage (%) relative to the standard deviation (RSD) were determined by UPLC–MS. The validation results are listed in Table 2. The RT of the compounds exhibited an excellent SD, regardless of the elution zones. Seven-point calibration curves were plotted over a 100-fold concentration range, and the linearities were excellent for mass detection (R > 0.995). The LOD and LOQ ranged from 0.4 to 93 μg/L and 1.3 to 308 μg/L, respectively (Table 2). The sensitivity ranged from 0.001 to 0.323 pmol/injection for mass detection (data not shown in Table 2). The sensitivity of mass detection was 10-fold that of the previous study [24]. The LODs of mass detection were lower (1–10-fold) than those reported for the determination of isoflavones using UPLC instrumentation [25]. The accuracy of recovery of the spiked compounds was within ±8% for mass detection, indicating suitable reliability. Intra- and interday precisions ranged from 1.3% to 8.3% RSD and 0.7% to 9.0% RSD (Table 2). These precisions were similar to those reported for UPLC-MS/MS instrumentation [26]. Despite the limitations of mass detection, compared to MS/MS instrumentation, our validation results indicate that the separation method is reliable for metabolomic analyses.

### 2.3. Changes in 31 Compounds in SLs

**Figure 2:**
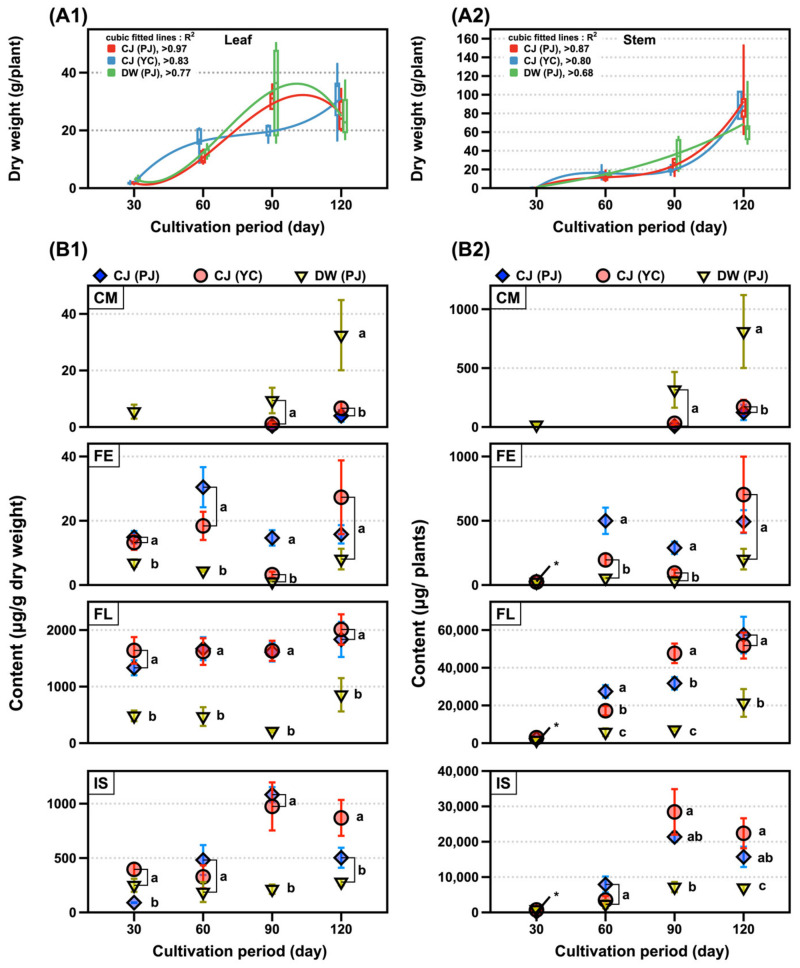
SB plant growth and changes of phenolics. The weight changes of (**A1**) leaf and (**A2**) stem and content of phenolic classes changes per (**B1**) dry weight of leaf and (**B2**) plant by cultivation period. Legends in figure: CJ, Cheng-ja no.3 variety; DW, Dae-won variety; PJ, Pa-ju city; YC, Yeon-chen city; CM, coumestan; FE, flavones; FL, flavonols; and IS, isoflavones. Lower case letters indicate statistical difference among the samples in the same cultivation period using comparisons of each pair by Student’s *t*-test (*p* < 0.05).

Three SB plants were grown in the field for over 120 days with similar growth patterns (Figure 2A1,A2). The dry weights of the SLs exponentially increased until the 90th day of growth, while those of the stems drastically increased after the 90th day of growth. Sixty samples of the SLs of two varieties in Pa-ju city (PJ) and one in Yeon-chen city (YC) were analyzed by UPLC–MS. The phenolic contents of the three SLs were compared across the cultivation periods (Figures S2 and S3). The isoflavone content doubled in 90 days of growth, compared with that of 30 days, while the flavonol content (approximately 2 mg/g) was two to three-fold higher across cultivation periods. Coumestrol was detected after 90 days and increased four-fold after 120 days; however, no significant changes were observed for the flavone content (Figure 2B1). Except for coumestrol, the contents of the other three compound classes in the SL of DW were lower than those in the two CJ (PJ and YC) (Figure 2B1). The isoflavone content slightly decreased after 90 days in the three SLs, while other polyphenol content steadily increased in the dry matter per leaf and per plant (Figure 2B1,B2). The maximum yield of the four classes of phenolics in the 90–120 day range was estimated as follows: coumestrol, 0.1–0.8 mg/plant; flavones, 0.2–0.7 mg/plant; flavonols, 21–57 mg/plant; and isoflavones, 7–28 mg/plant (Figure 2B2 and Table S2).

**Figure 3:**
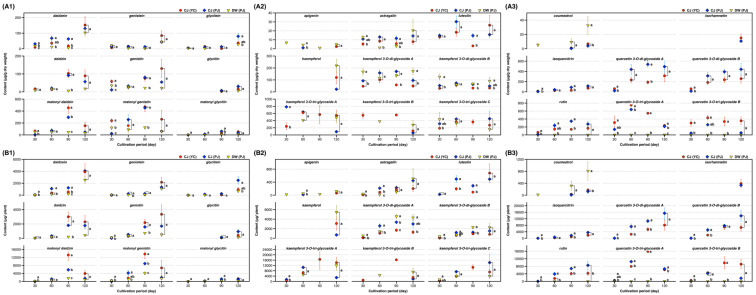
Changes of individual compounds in the SB leaves by cultivation periods. Content per (**A**) dry weight of the leaves and (**B**) plant; (**A1** and **B1**) isoflavones, (**A2** and **B2**) flavones and kaempferol glycosides, and (**A3** and **B3**) coumestrol, isorhamnetin, and quercetin glycosides. Legends in figure: CJ, Cheng-ja no.3 variety; DW, Dae-won variety; PJ, Pa-ju city; and YC, Yeon-chen city. Lower case letters indicate statistical difference among the samples in the same cultivation period using comparisons of each pair by Student’s *t*-test (*p* < 0.05).

The changes in the 31 compounds across the cultivation period are shown in Figure 3 and Table S3. Malonyl daidzin and malonyl genistin were dominant (approximately 60%) among the 12 isoflavones, while glycitein and its conjugates were the least abundant. Isoflavones largely increased across the cultivation period; however, malonyl isoflavones decreased, and isoflavone aglycones increased after 120 days. This might be due to changes in the biosynthesis flux in the mature SB plant [2]. The SL of DW possessed a small amount of each isoflavone compared to that of the SL of CJ (Figure 3A1). Kaempferol triglycosides constituted a major portion of the kaempferol derivatives. Kaempferol derivatives including aglycone, mono, di, and triglycosides were commonly detected in three SLs, while luteolin was observed in only the SL of CJ in trace amounts (approximately 20 µg/g dry weight) (Figure 3A2). Remarkably, quercetin derivatives were present in the SL of CJ, which is considered a distinctive characteristic of the black-coat SB [22]. Only neglectable amounts of quercetin triglycosides were observed in the SL of DW, and considerable amounts of quercetin di and triglycosides were present in SL of CJ and increased across the cultivation period (Figure 3A3). Coumestrol was detected after 90 and 120 days, which is a well-known phenomenon that occurs because of endogenous tolerance against stress in a mature SB plant [27,28]. The maximum yield of the remarkable individual compounds within 90–120 days were estimated as follows: daidzin, 3 mg/plant; genistin, 3 mg/plant; kaempferol 3-*O*-di-glycoside A, 5 mg/plant; kaempferol 3-*O*-tri-glycoside A, 17 mg/plant; kaempferol 3-*O*-tri-glycoside C, 14 mg/plant; malonyl daidzin, 13 mg/plant; malonyl genistein, 9 mg/plant; rutin, 9 mg/plant; quercetin 3-*O*-di-glycoside A, 15 mg/plant; and quercetin 3-*O*-di-glycoside B, 14 mg/plant (Figure 3B and Table S4).

The changes in the SL flavonoids can be affected by environmental factors, such as soil composition and climate. Flavonoid synthesis can be modulated through protective functions, regulation of gene expression, and nutritional necessity of plants against environmental, abiotic/biotic, and nutritional stresses, respectively (e.g., UV radiation and reactive oxygen species, activation and repression, and metal chelating, respectively) [29]. Therefore, a comparative study must be conducted with firmly controlled cultivation conditions. Furthermore, agricultural products exhibit wide variation (20–40% RSD) in phenolic composition, even if the conditions are well controlled [30]. Despite the difficulties of a study that handles field-grown plants, secondary metabolites are indisputable markers originating from inherent genetic traits [31]. Nontargeted metabolomics is a prominent technology with sophisticated instrumental manipulations to understand the differences in genetic diversity via secondary metabolites [15,32]. However, there are many barriers and prerequisites for delicate advanced analyses. For example, expensive operation, consolidated analysis process accompanying QC and QA samples, and considerable numbers of manual peak identification [33]. While the latest advanced nontargeted analysis seems to improve understanding for metabolome in a wide range, the targeted method could be a practical approach to interpret and build a reproducible prediction model with moderate numbers of metabolic markers accompanied by lesser time and resource requirements [12].

### 2.4. Supervised ML Model Predictions via Targeted Metabolomics

We obtained a reliable quantification summary using the aforementioned post data processing, and an unsupervised PCA overview was obtained from an organized data set regarding the differences in the targeted compounds for cultivation days, cities, and varieties using SIMCA 17 (Umetrics, Umeå, Sweden). In the PCA plot (Figure S16), the first and second principal components elucidated 34% and 19% of the variation, respectively. The fitness of the established PCA model was 0.64 (R^2^) and 0.33 (Q^2^), and the score plot exhibited non- or weak clustering by days and varieties. To discriminate the differences in the targeted chemical compositions, supervised orthogonal partial least-squares (OPLS) regression and OPLS-DA were used.

**Figure 4:**
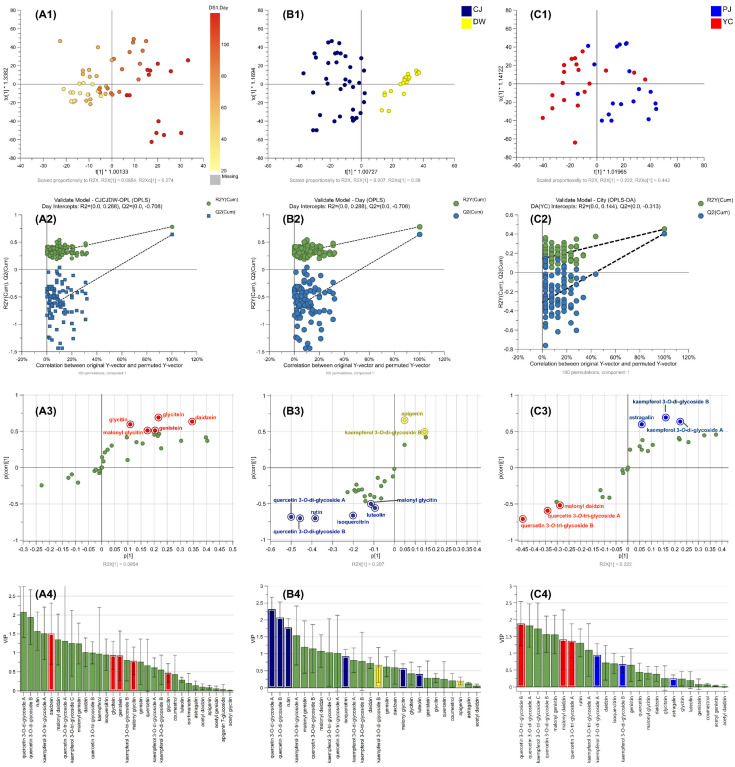
Multivariate analysis of SL metabolites. (**A1**) OPLS score plot with one y-variable (cultivation day), (**A2**) validation of the OPLS model using 100-permutation test, (**A3**) S-plot of OPLS, and (**A4**) VIP plot, (**B1** and **C1**) OPLS-DA score plots for variety of SB plant and cultivation sites, (B2 and C2) validation of the models using 100-permutation, (**B3** and **C3**) S-plot of OPLS-DA, and (**B4** and **C4**) VIP plots.

The OPLS regression model for discriminating the difference by cultivation days was established, and the score plot indicated that metabolome differences can be explained by days (left to right) (Figure 4A1). The values of R^2^X, R^2^Y, and Q^2^ of the OPLS regression model were 0.81, 0.78, and 0.64, respectively, indicating relatively suitable fitness (Table S5) [34]. The established model was considered reliable according to the cross-validation with a 100-permutation test. Green R^2^- and blue Q^2^-values to the left were lower than the original points to the right, and the regression line of the Q^2^-points intersected the vertical axis below zero (−0.708) (Figure 4A2). In the S-plot, p of *x*-axis and p _(corr)_ of *y*-axis represent a contribution (covariance) of the compounds to the variance of the observations and correlation between samples and the reliability of the results. The metabolites within our criteria of |p| ≥ 0.05 and |p_(corr)_| ≥ 0.5 in the S-plot were highlighted (Figure 4A3), and the corresponding compounds were depicted in the same color in the variable importance projection (VIP) plot (Figure 4A4). The highlighted red dots in the S-plot indicate the metabolites that increased during the cultivation days. Five isoflavones were listed as important metabolites to discriminate the cultivation days (Table 3 and Table S6).

The OPLS-DA model for discriminating the difference by variety was formulated and produced a score plot that was well clustered between the varieties (Figure 4B1). The model showed relatively suitable fitness values (R^2^X, 0.60; R^2^Y, 0.80; and Q^2^, 0.73; Table S5) and was considered valid based on the 100-permutation test (Figure 4B2; intersect of Q^2^, −0.386). Four key metabolites were screened based on our cut-off criteria using the S-plot and VIP values (Table 3, Table S6,S7). All the discriminative compounds were flavonol glycosides that originated from the CJ variety (isoquercitrin, quercetin diglycosides, and rutin) (Figure 4B3).

When a targeted metabolomic approach is conducted using a limited number of subject compounds, there is inadequate information to discriminate the characteristics. However, if statistically sufficient numbers of the compounds of interest are determined, it is useful and applicable for estimating the compound content in certain agricultural products using ML methods [35]. The benefits of these targeted metabolomic analyses can be expanded to establish a reliable predictive model for tracking an object to be considered. The ML method is a prominent and unique tool for understanding scattered information, even for small data sets [35]. We surmised that our data set of 60 samples for quantitative results of 31 compounds was adequate for application following the ML prerequisite.

**Figure 5:**
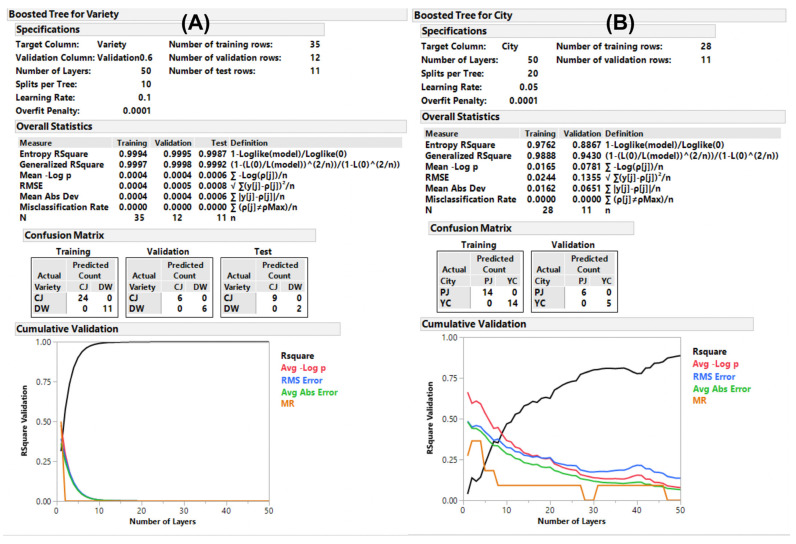
Predictive modeling using machine learning. Reports of the boosted tree model for (**A**) the variety and (**B**) the cultivate on-site discriminations.

The organized data set used in the aforementioned OPLS and OPLS-DA was randomly divided into three sub-data sets (training, validation, and test), and the predictions were computed using neural (NU), bootstrap forest (BF), and boosted tree (BT) models using JMP 13 pro (SAS Institute Inc., Cary, NC, USA). The classes to be discriminated were varieties (CJ and DW), days (30, 60, 90, and 120), and cities (PJ and YC). The models established using NU exhibited suitable fitness (generalized R^2^ ranged from 0.94 to 0.99) in the three sub-data sets with multiple layers of hidden computation (Table S5D). The fitness of the models established using BF and BT differed by comparison classes. The BF model discriminated multiple components of a class, while the BT model was effective on a two-component class of the data set to be analyzed. Accordingly, the cultivation days were effectively discriminated using the BF model, which was not applicable to the BT model. The classes of variety and city showed lower fitness in the BF model (data not shown) compared to that of BT. The BT model for the variety displayed excellent fitness (>0.99) for all three sub-data sets (Figure 5 and Table S5D). The comparison of cities using the BT model was not applicable to the test set because of inadequate effective data; therefore, the model was established without it. The key compounds obtained using the BF and BT methods are listed in Table S8 using the criteria (sum of contributing portion of G^2^ > 90% in the order of the portion). None were obtained using NU because of the hidden node decision feature. Compared to the OPLS and OPLS-DA outputs of the key compounds, the other ML methods included slightly different compounds. However, we observed that there were similarities in the compounds listed for the supervised ML methods; therefore, we created combined criteria for the key compounds derived from the discriminative classes.

The key compounds screened using the combined criteria are listed in Table 3. Certain isoflavones were discriminators for growth (days in Table 3), certain quercetin derivatives and flavones for variety, and certain flavonol derivatives for cultivation sites. The model established by ML of JMP pro can be exported as a coded function to be used without statistical software (Supplementary Data II). We embedded the executable Python file with six predictions derived from three ML methods of JMP pro (Script S1) and presented an example result of the ML prediction for the test data set. A total of 1 or 2 of the 10 were misclassified in the day class discrimination, and all predictions were the same as the originals in the other class discriminations (Table S9). In addition, the OPLS-DA of SIMCA can generate a misclassification rate with similar prediction power to ML of JMP pro; however, we did not demonstrate using SIMCA because it can be obtained via the software. The prediction power of NU appeared excellent across all the ML methods with high fitness and low misclassification rates (Table S5D and Figure S17). The decision tree learning method presented in our study may potentially be applicable to discriminate an influence of agricultural factors using plant flavonoids of interest even with a small data set that is segmented many classes of sub-factors.

NU model implements a fully connected multi-layer perceptron with one or two layers. The main advantage of a neural network model is that it can efficiently model different response surfaces. Given enough hidden nodes and layers, any surface can be approximated to any accuracy. The main disadvantage of a neural network model is that the results are not easily interpretable. BF model predicts a response value by averaging the predicted response values across many decision trees. BT model embedded a boosting process of which building a large, additive decision tree by fitting a sequence of smaller decision trees. For categorical responses, only those with two response levels are supported by BT [36]. Consequently, considerations must be taken for a relatively high overfitting risk, sample numbers, and collinearity of data in the use of NU, BF (or BT), and OPLS methods, respectively [20].

## 3. Materials and Methods

### 3.1. Chemicals and Reagents

Apigenin, apigenin 7-*O*-glucoside, daidzein, daidzin, daidzin 6″-*O*-acetate, daidzin 6″-*O*-malonate, genistein, genistin, genistin 6″-*O*-acetate, genistin 6″-*O*-malonate, glycitein, glycitin, glycitin 6″-*O*-acetate, and glycitin 6″-*O*-malonate were purchased from Fujifilm Wako Pure Chemical Industries, Ltd. (Osaka, Japan). Coumestrol, dimethyl sulfoxide (DMSO), kaempferol, kaempferol 3-*O*-glucoside (astragalin), isoquercitrin, isorhamnetin, luteolin, quercetin, and rutin were purchased from Sigma-Aldrich Co., LLC (St. Louis, MO, USA). The purities of the authentic compounds are listed in Table S1. Two quercetin diglycosides, two quercetin triglycosides, two kaempferol diglycosides, and three kaempferol triglycosides were purified from SLs. Mass-grade formic acid, acetonitrile, methanol, and water were purchased from Thermo Fisher Scientific Inc. (Waltham, MA, USA). Other chemicals used were of American Chemical Society grade or higher.

### 3.2. SB Seeding, Cultivation, and Leaf Sample Preparation

Two SB plant varieties, CJ and DW, for metabolomic analysis and two other varieties, NP and WR, for purified flavonol glycosides, were provided by the National Institute of Crop Science, Korea (CJ, DW, and WR) and Gyeonggi-do Agricultural Research & Extension Services, Korea (NP). CJ possesses a black coat and green cotyledon, and DW has been widely cultivated for over 30 years with a creamy yellow coat and cotyledon. CJ was cultivated in YC, Gyeonggi Province, Korea (37.923161° N, 126.726393° E). Simultaneously, CJ and DW were cultivated in PJ, Gyeonggi Province, Republic of Korea (38.082867° N, 127.075570° E). Each variety was planted in the first week of June 2018 within a 330 square meters area. The seeding method followed the standards of the National Institute of Crop Science [37]. The seeds were contacted in a fungicidal agent (mixed wettable powder of benomyl (20%; *w*/*w*) and thiram (20%; *w*/*w*); at a 4 g/kg seed), and two seeds were sown in a single spot with a density of 700 × 150 mm (field ridge × row). Bacterial inoculants were not used. The methods of topdressing and cultivation followed an authentic manual without pesticide treatment during growth. After planting, five whole plants were randomly uprooted every 30, 60, 90, and 120 days of growth. The plucked samples were washed with deionized water, and the leaves were collected without stems. The moisture of the leaves was removed using a FreeZone™ freeze dryer (Labconco Corp., Kansas City, MO, USA). The dried leaves were ground using a Tubemill™ (IKA^®^-Werke GmbH & Co. KG, Staufen, Germany) at 25,000 rpm for 1 min, placed in aluminum-laminated polyethylene packaging, and stored at −20 °C until use.

### 3.3. Preparation and Identification of Flavonol Glycosides from SLs

To secure flavonol glycosides from SLs, the aforementioned varieties were grown for 90 days. The uprooted SB plants were treated as previously described. Each SL powder was soaked in 60% (*v*/*v*) aqueous methanol (50 mg/mL) for 15 h at 25 °C. The methanolic extract solutions were centrifuged at 4000× *g* for 10 min, and the supernatants were filtered through a 0.45 µm poly vinylidene fluoride (PVDF) syringe filter (Pall Inc., Port Washington, NY, USA). The filtered solutions were injected (200–1000 µL per injection) in a preparative HPLC column equipped with a 172-diode array detector, 321-binary pump, and GX-271 liquid handler (Gilson Inc., Middleton, WI, USA). Each flavonol glycoside was fractionated for a single peak under programed elution conditions using a preparative separation column (ZORBAX Eclipse XDB C_18_, 80 Å, 5 µm, 21.2 × 150 mm, Agilent Technologies Inc. Santa Clara, CA, USA) at 30 °C with a Jasco CO-2060 column heater (Tokyo, Japan). The collected fractions in repeated injections were combined, evaporated, and freeze-dried. The details of the preparative elution programs and peak collection are shown in Figure S1.

The purified flavonol glycosides were identified using high-resolution mass spectrometry (HRMS). An UltiMate 3000 (Thermo Scientific Inc., Waltham, MA, USA) with a Waters Cortecs C_18_ column (90 Å, 1.6 um, 2.1 × 100 mm, Milford, MA, USA) was used for separation. The column temperature was set at 40 °C, and the flow rate was 0.5 mL/min. The mobile phase comprised 0.1% (*v*/*v*) formic acid in water (solvent A) and 0.1% (*v*/*v*) formic acid in acetonitrile (solvent B). A linear gradient was applied as follows: 86% A/14% B at 0 min, 86% A/14% B at 1.5 min, 74% A/26% B at 3 min, 74% A/26% B at 5 min, 20% A/80% B at 5.5 min, 86% A/14% B at 6 min, and 86% A/14% B at 7 min. The mass detection of precursors and fragments was performed using triple TOF 5600+ (AB Sciex LLC., Framingham, MA, USA) under the following conditions: ionization mode, positive and negative; MS scan type, full scan, and information-dependent acquisition (IDA) scanning; ionization source, ESI; MS scan range, 200–2000 mass-to-charge ratio (*m*/*z)*; MS/MS scan range, 30–2000 *m*/*z*; nebulizing gas pressure (ion source 1), 50 psi; heating gas pressure (ion source 2), 50 psi; curtain gas pressure, 25 psi; desolvation temperature, 500 °C; ion spray voltage floating, 5.5 kV (positive) and 4.5 kV (negative); declustering potential (DP), 60 (positive) and −60 (negative); collision energy (CE), 10 (positive) and −10 (negative); collision energy, 35 ± 15 (positive) and −35 ± 15 (negative); collision gas, N_2_.

The purity of the compounds was tentatively calculated by spectral purity check using an ACQUITY PDA detector and Empower 3 software (Waters Corp.) [38,39]. Two kaempferol diglycosides and one kaempferol triglycoside, two kaempferol triglycosides, two quercetin diglycosides, and two quercetin triglycosides were separated from WR, DW, CJ, and NP, respectively.

### 3.4. Analytical Conditions for the Quantification of the 31 Compounds

An ACQUITY UPLC (Waters Corp.) equipped with a binary pump and the Cortecs C_18_ column was used for the separation. The column temperature was 30 °C, and the injection volume was 1 µL. Other separation conditions were as previously described. The eluent was passed through the ACQUITY PDA detector and an ACQUITY QDa™ MS.

The MS was optimally tuned in the range of critical parameters: capillary voltage (0.4–0.8 Kv) and cone voltage (5–30 V). Mass detection was performed using the following parameters: capillary voltage, 0.8 kV; probe temperature, 600 °C; ESI source temperature, 120 °C; and desolvation nitrogen gas pressure, 90 psi. Cone voltages were allocated for the chemicals: isoflavones, 5 V and others, 15 V. A single ion recording was performed in positive mode for isoflavones and negative mode for others. The mass data of the compounds analyzed were acquired (5 points/s) within a time window of ±8.70–24.3 s based on the RT of the compounds (Table S1). All data were collected and processed using Empower 3 (Waters Corp.).

### 3.5. Stock Solutions and SL Sample Preparations

Fresh stock solutions containing 31 compounds (12 isoflavones, 1 coumestrol, 9 flavonoid reagents, and 9 purified flavonol glycosides) were prepared by mixing 2000 mg/L of each compound in DMSO. Working solutions were prepared by diluting the stock solutions with similar initial elution solutions (0.1% (*v*/*v*) formic acid in 20% aqueous acetonitrile).

Ground SL (10 mg) was added to 60% (*v*/*v*) aqueous methanol (5 mL) and sonicated for 20 min. The supernatant obtained by centrifugation at 4000× *g* for 10 min was filtered through a 0.2 µm regenerated cellulose Claristep^®^ syringeless filter (Sartorius, Göttingen, Germany). Thereafter, it was properly diluted with 0.1% formic acid in 20% acetonitrile in the range of 100–200 mg/L for quantitative analysis.

### 3.6. Separation Method Validation

A mixed solution of 31 standard compounds (0.1 mg/L) was injected into the system 10 times to calculate the LOD, LOQ, and system suitability. The validation of the UPLC–MS methods was performed with the acquisition of the same injection regarding intraday precision, interday precision, linearity, LOD, LOQ, and accuracy. The overall method validation rules described in “Eurachem Guide: The Fitness for Purpose of Analytical Methods, 2nd Edition 2014” were followed [40]. The quadratic quantification curves of the 31 compounds were prepared by the injection of 7-point mixed standard solutions within a concentration range of 0.2–20.0 mg/L.

The quantification data for the samples were expressed as the means ± standard errors of the mean based on the five samples. One-way analysis of variance was performed using comparisons of each pair by Student’s *t*-test with the *p* < 0.05, using JMP 13 pro.

### 3.7. Metabolomic Discrimination Using ML Methods

The area under the peaks of the targeted 31 compounds presented in standards and samples were obtained after a smoothing treatment of the original peak using the mathematical mean method (level 15) by Empower 3 (Waters Corp.). Thereafter, the processed results were exported to a .csv file, and the content of the compounds was calculated using a coded algorithm similar to that in a previous study [41]. The algorithm for quantifying and eliminating unnecessary or unmatched data was implemented using R software 4.1.1 (The R Foundation, Vienna, Austria) and RStudio 1.4.1717 (RStudio, Boston, MA, USA).

To perform OPLS and OPLS-DA, additional information of the analyzed samples was added to the acquired data set of the cultivation days of 30, 60, 90, and 120 (regression model) and varieties and cities (discriminant model). Zero values were entered for the blank quantification data (undetected or under the LOQ). The organized data set was implemented by SIMCA and was centering with pareto scale (Par), including OPLS regression and OPLS-DA. The implemented data set was not classified into validation and test set due to the lack of numbers of data for OPLS and OPLS-DA. The models were fitted using the “Autofit” function of SIMCA and were then optimized by eliminating outliers that were located far outside in the score plot and residual normal probability plot until the highest R^2^ and Q^2^ values of the fitted models were obtained. An S-plot and VIP were employed to screen compounds that were responsible for the discrimination of the class to be compared. The cut-off combined criteria were set as |p| ≥ 0.05 and |p_(corr)_| ≥ 0.5 for the S-plot and VIP > 0.8 [33,42,43]. A cross-validation of the OPLS regression and OPLS-DA model was performed using a 100-permutation test for the optimized data set performed using SIMCA 17.

The organized data set prepared was imported to JMP 13 pro to select key compounds for discrimination and to examine the corresponding prediction power using ML methods, including NU, BF, and BT. The imported data set was randomly classified into training, validation, and test sets in portions of 0.6, 0.2, and 0.2, respectively, using tools in the software. Some parameters of these models were tested in discrete numbers to find an optimal result. The following bold numbers indicate the optimal options among them. Otherwise, default numbers in the option were chosen. The NU model was launched with the following options: hidden layer structure, (TanH (1, 2, **3**), number of models (1, 5, **10**), and learning rate (**0.05**, 0.1)) and fitting options, (penalty method, absolute, and number of tours (1, 5, 10)). The BF model was launched with the following options: forest, (number of trees in the forest (10, 50, 100), number of terms sampled per split (6), bootstrap sample rate (10), minimum splits per tree (10), maximum splits per tree (2000), and minimum size split (5)) and multiple fits, not checked. The BT model was launched with the following options: number of layers (10, 20, **50**); split per tree, 3; learning rate, (**0.05**, 0.1); overfit penalty, 0.0001; and minimum size split, 5. Statistics and misclassification rates were checked for the prediction power of the three ML methods of JMP pro and listed for the compounds with the highest contribution to the established model. The formulas of the prediction models were exported to Python code and applied to the test data set for discriminating certain attributes using PyCharm (JetBrains sro, Prague, Czech Republic) environment.

The key compounds for discriminating the classes (days, varieties, and cities) were selected using the combined criteria of the four values from the ML methods. No concrete rules for cut-off criteria had not been suggested [44,45]. We use the commonly used limit for p and p_(corr)_ [33] and for VIP [43] as follows. At least two conditions of the following three have to be satisfied: (1) |p| ≥ 0.05 and |p_(corr)_| ≥ 0.5 for the S-plot of OPLS and OPLS-DA, (2) VIP > 0.8 for the OPLS and OPLS-DA, and (3) sum of contributing portion of G^2^ > 90% of ML methods. G^2^ is a fit statistic used for categorical responses instead of the sum of squares used for continuous responses, where a nonzero G^2^ value indicates a splitting possibility in the decision tree [46].

## 4. Conclusions

Various chemometric, ML, and targeted metabolomic analyses have been applied to diverse food and agricultural products [19,35,47,48]. Recently, these methods have been used to discover concrete correlations between antioxidants and their efficacies [41,49]. We presented changes in 31 targeted compounds and key compounds in SLs using ML predictions across the variety, growth, and cultivation sites of the SB plant. Regarding predictability, NU is a suitable predictor or discriminator for various confusing matters, such as product quality, origin distinction, and production yields. The SL is a useful agricultural resource for the phytochemical use of value-adding bioactive compounds. In addition, the phenolic compounds of SL exhibit health-promoting effects [50,51,52]. Further metabolomic studies should be performed on diverse SLs and various cultivation environmental factors. This approach provides valuable clues for discriminating more diverse characteristics of food and agricultural products.

## Figures and Tables

**Table 1:** Mass spectral data of purified flavonol glycosides from various soybean leaves.

Compound ^†^	Positive			Negative				Tentative Identification
	MS1 ^a^	Error ppm	MS2 ^b^	MS1 ^a^	Error ppm	MS2 ^b^	Formula, (M)	
CJ1	773.2120	−0.2	303.0489, 465.1016, 611.1591, 627.1523	771.2016	−0.1	301.0372	C_33_H_40_O_21_	quercetin 3-*O*-tri-glycoside A
CJ2	773.2133	0.1	303.0497, 465.1022, 611.1601, 627.1552	771.2041	0.5	301.0374	C_33_H_40_O_21_	quercetin 3-*O*-tri-glycoside B
DW1	757.2162	−0.4	287.0542, 449.1076, 595.1634 611.1558	755.2048	−0.3	285.0400	C_33_H_40_O_20_	kaempferol 3-*O*-tri-glycoside A
DW2	757.2173	0.1	287.0539, 449.1071, 595.1638 611.1597	755.2040	−0.5	285.0408	C_33_H_40_O_20_	kaempferol 3-*O*-tri-glycoside B
NP1	627.1535	0.3	303.0494, 465.1018	625.1466	−0.4	301.0372	C_27_H_30_O_17_	quercetin 3-*O*-di-glycoside A
NP2	627.1539	−0.6	303.0489, 465.1012	625.1473	−0.1	301.0365	C_27_H_30_O_17_	quercetin 3-*O*-di-glycoside B
WR1	741.2218	−0.4	287.0541, 449.1070, 595.1646	739.2132	0.0	285.0331	C_33_H_40_O_19_	kaempferol 3-*O*-tri-glycoside C
WR2	595.1634	−0.1	287.0553, 449.1067	593.1540	0.2	285.0414	C_27_H_30_O_15_	kaempferol 3-*O*-di-glycoside A
WR3	595.1628	−0.3	287.0549, 449.1060	593.1545	0.2	285.0416	C_27_H_30_O_15_	kaempferol 3-*O*-di-glycoside B

^†^ Compounds from CJ, Cheng-ja; DW, Dae-won soybean variety; NP, Nok-poong; and WR, Woo-ram varieties. ^a^ Molecular mass of precursors. ^b^ Molecular mass of molecular fragments (Refer to the details in Figures S2–S5).

**Table 2:** Method validation of mass detection for 31 compounds presented in soybean leaf.

No. ^a^	CL ^b^	Compound	RT ^c^ (min)	LOD ^d^ (μg/L)	LOQ ^e^ (μg/L)	Linearity (R ^f^)	Accuracy (Recovery % ± SEM) ^g^	Precision (RSD; %) ^h^	
								Intraday ^i^	Interday ^j^
1	FL	quercetin 3-*O*-tri-glycoside A	1.04 ± 0.002	8.21 ± 0.71	27.35 ± 2.35	0.9997	102.14 ± 1.24	3.84	3.17
2	FL	quercetin 3-*O*-tri-glycoside B	1.09 ± 0.001	1.23 ± 0.23	4.11 ± 0.77	0.9971	95.70 ± 2.66	8.32	5.35
3	FL	quercetin 3-*O*-di-glycoside A	1.34 ± 0.002	5.48 ± 0.57	18.26 ± 1.89	0.9998	93.96 ± 1.59	5.35	4.83
4	FL	quercetin 3-*O*-di-glycoside B	1.40 ± 0.002	8.12 ± 1.58	27.07 ± 5.27	0.9995	100.92 ± 1.52	4.76	2.89
5	IS	daidzin	1.45 ± 0.002	4.98 ± 0.32	16.61 ± 1.07	0.9991	108.81 ± 0.45	1.31	3.53
6	FL	kaempferol 3-*O*-tri-glycoside A	1.48 ± 0.002	38.09 ± 1.64	126.97 ± 5.46	0.9997	105.62 ± 0.62	1.87	6.50
7	FL	kaempferol 3-*O*-tri-glycoside B	1.62 ± 0.002	57.03 ± 2.53	190.10 ± 8.44	0.9991	102.07 ± 2.61	8.09	8.97
8	IS	glycitin	1.67 ± 0.002	17.53 ± 1.41	58.43 ± 4.71	0.9992	104.96 ± 1.40	4.23	3.44
9	FL	kaempferol 3-*O*-tri-glycoside C	2.01 ± 0.002	8.26 ± 0.97	27.55 ± 3.25	0.9996	103.88 ± 1.07	3.26	2.50
10	FL	rutin	2.45 ± 0.001	5.81 ± 0.40	19.38 ± 1.33	0.9998	101.57 ± 1.00	3.11	3.35
11	FL	isoquercitrin	2.61 ± 0.001	3.58 ± 0.16	11.94 ± 0.52	0.9997	104.43 ± 0.81	2.45	3.43
12	IS	genistin	2.70 ± 0.000	14.76 ± 0.81	49.19 ± 2.71	0.9995	102.51 ± 1.03	3.18	2.97
13	FL	kaempferol 3-*O*-di-glycoside A	2.72 ± 0.000	2.64 ± 0.13	8.80 ± 0.45	0.9997	100.51 ± 1.12	3.51	1.49
14	FL	kaempferol 3-*O*-di-glycoside B	2.91 ± 0.000	7.09 ± 0.10	23.65 ± 0.33	0.9992	96.73 ± 1.07	3.48	4.42
15	IS	malonyl daidzin	2.92 ± 0.000	13.12 ± 0.80	43.72 ± 2.66	0.9997	102.90 ± 1.25	3.85	3.70
16	IS	malonyl glycitin	2.98 ± 0.000	5.55 ± 0.52	18.49 ± 1.72	0.9998	100.27 ± 1.18	3.71	4.65
17	FL	astragalin	3.03 ± 0.000	1.57 ± 0.36	5.23 ± 1.21	0.9995	103.24 ± 0.61	1.86	3.62
18	FE	apigenin 7-*O*-glucoside	3.15 ± 0.000	0.38 ± 0.07	1.27 ± 0.23	0.9981	106.70 ± 1.36	4.04	2.38
19	IS	acetyl daidzin	3.29 ± 0.000	5.45 ± 0.25	18.16 ± 0.84	0.9993	101.76 ± 0.86	2.68	2.45
20	IS	acetyl glycitin	3.38 ± 0.000	2.46 ± 0.03	8.20 ± 0.09	0.9997	102.34 ± 0.51	1.56	2.15
21	IS	malonyl genistin	3.45 ± 0.000	8.52 ± 0.03	28.39 ± 0.09	1.0000	99.12 ± 0.44	1.40	2.14
22	IS	daidzein	3.79 ± 0.000	7.87 ± 0.15	26.22 ± 0.50	0.9995	100.31 ± 0.94	2.98	2.61
23	IS	acetyl genistin	3.97 ± 0.000	12.03 ± 0.18	40.10 ± 0.61	0.9995	100.90 ± 0.95	2.99	2.76
24	IS	glycitein	4.01 ± 0.000	18.12 ± 0.35	60.40 ± 1.18	0.9995	100.45 ± 0.71	2.24	1.52
25	FE	luteolin	4.19 ± 0.001	7.76 ± 0.22	25.88 ± 0.75	0.9998	101.01 ± 0.81	2.53	2.27
26	FL	quercetin	4.21 ± 0.000	9.94 ± 0.38	33.14 ± 1.26	0.9995	101.08 ± 0.67	2.09	2.30
27	IS	genistein	5.40 ± 0.001	45.18 ± 2.02	150.59 ± 6.73	0.9998	100.92 ± 0.43	1.35	0.70
28	FE	apigenin	5.52 ± 0.001	15.20 ± 0.87	50.65 ± 2.90	0.9958	101.30 ± 1.21	3.79	3.90
29	CM	coumestrol	5.58 ± 0.000	2.16 ± 0.19	7.19 ± 0.63	0.9965	105.20 ± 0.60	1.80	2.24
30	FL	kaempferol	5.65 ± 0.000	92.49 ± 10.43	308.29 ± 34.78	0.9992	104.37 ± 0.68	2.05	3.73
31	FL	isorhamnetin	5.71 ± 0.000	35.14 ± 3.14	117.12 ± 10.47	0.9990	108.85 ± 1.19	3.45	5.41

^a^ Numbered in the order of retention time (RT). ^b^ Class: CM, coumestan; FE, flavone; FL, flavonol; and IS, isoflavone. ^c^ Retention time (RT) was presented the mean ± standard error of the means (*n* = 10). ^d^ Limit of detection. ^e^ Limit of quantification. ^f^ Correlation coefficient. ^g^ Trueness includes an accuracy term (*n* = 6), with 95% confidence interval. ^h^ RSD: relative standard deviation. ^i^ Intraday variation of analysis (*n* = 6). ^j^ Interday variation of analysis (*n* = 6 × 3 days).

**Table 3:** Key phenolic compounds for cultivation period, varieties, and cultivation sites.

Class of Data Set ^a^	Compound ^b^	OPLS or OPLS-DA ^c^			BF or BT ^g^	
		p ^d^	p _(corr)_ ^e^	VIP ^f^	G^2 h^	Portion ^i^
Day	daidzein	0.345	0.636	1.51	5.00	0.14
	genistein	0.203	0.512	LL ^j^	4.82	0.13
	glycitein	0.217	0.696	LL	7.96	0.22
	malonyl glycitin	0.174	0.512	0.79	6.05	0.17
Variety	apigenin	0.049	0.656	LL	581.90	0.27
	luteolin	−0.095	−0.556	LL	959.18	0.44
	isoquercitrin	−0.200	−0.661	0.92	LL	LL
	quercetin 3-*O*-di-glycoside A	−0.500	−0.688	2.31	LL	LL
	quercetin 3-*O*-di-glycoside B	−0.457	−0.705	2.07	LL	LL
	rutin	−0.386	−0.703	1.77	LL	LL
	quercetin 3-*O*-tri-glycoside A	LL	LL	1.02	593.46	0.27
City	astragalin	0.060	0.596	LL	17.81	LL
	kaempferol 3-*O*-di-glycoside A	0.221	0.633	0.94	258.71	0.12
	malonyl daidzin	−0.290	−0.519	1.41	LL	LL
	quercetin 3-*O*-tri-glycoside A	−0.340	−0.597	1.37	68.08	LL
	quercetin 3-*O*-tri-glycoside B	−0.448	−0.705	1.88	183.69	0.08

^a^ Day, (30, 60, 90, and 120 days of cultivation period); variety, (CJ of PJ city, CJ of YC city, and DW of PJ city); and city, only CJ variety was compared in the two cities. ^b^ Data were filtered, which satisfied at least two conditions among three. ^c^ OPLS, orthogonal partial least-squares; and OPLS-DA, orthogonal partial least-squares discriminant analysis. ^d^ Modeled covariation. ^e^ Correlation coefficient. ^f^ Variable importance projection. ^g^ BF, bootstrap forests; and BT, boosted tree. ^h^ Likelihood ratio chi-square. ^i^ The portion is among the compounds that have G^2^ value according to the result of machine learning modeling. ^j^ LL: lower than the criteria (|p| ≥ 0.05, |p_(corr)_| ≥ 0.5, VIP ≥ 0.8, and within sum of portion of G^2^ > 90%).

## Data Availability

The executable Python codes for the predictions can be found in doi:10.17632/4cxz6d7ymw.1.

## Supplementary Materials

The following are available online at https://www.mdpi.com/article/10.3390/metabo11120884/s1, Tables S1–S9, Figures S1–S17, and Script S1 showing detail are presented in Supplementary Data I; method validation, quantifications, detailed information for the purified flavonol glycosides, multivariate analysis, modeling reports of machine learning, and Python code for predictions. Supplementary Data II for the executable Python codes for the predictions can be found in doi:10.17632/4cxz6d7ymw.1.
