# Supplementary material for: Supervised Statistical Learning Prediction of Soybean Varieties and Cultivation Sites Using Rapid UPLC-MS Separation, Method Validation, and Targeted Metabolomic Analysis of 31 Phenolic Compounds in the Leaves
Source: Metabolites. 2021 Dec 17;11(12):884. doi: 10.3390/metabo11120884 (PMC8704512; doi:10.3390/metabo11120884)
Supplement: Supplementary file 1 [file metabolites-11-00884-s001.zip › metabolites-1492248-supplementary.pdf]

Article

Supervised Statistical Learning Prediction of Soybean Varieties and Cultivation Sites Using Rapid UPLC-MS Separation, Method Validation, and Targeted Metabolomic Analysis of 31 Phenolic Compounds in the Leaves

Chan-Su Rha [1,*], Eun Kyu Jang [2], Yong Deog Hong [1] and Won Seok Park [1]

[1] AMOREPACIFIC R&D Center, Yongin 17074, Korea; hydhong@amorepacific.com (Y.D.H.);
wspark@amorepacific.com (W.S.P.)
[2] Gyeonggi-do Agricultural Research & Extension Services, Hwaseong 18388, Korea; jek0428@gg.go.kr
* Correspondence: teaman@amorepacific.com; Tel.: +82-(31)-2805981

Supplementary Materials

Table of Contents

Tables

Table S1. Purity of compounds used in method validation and conditions of mass detection................ 3

Table S2. Content of phenolic classes in the soybean leaves by cultivation days.................................. 4

Table S3. Content of phenolic compounds in soybean leaves per dry weight. 5

Table S4. Content of phenolic compounds in soybean leaves per plant. 7

Table S5. Summary of machine learning models... 9

Table S6. Key compounds obtained using cut-off criteria of S-plot (SIMCA).................................... 10

Table S7. Key compounds obtained using cut-off criteria of VIP (SIMCA) 11

Table S8. Key compounds obtained using cut-off criteria of machine learning (JMP pro)................. 12

Table S9. Example of prediction results executed by Python code... 13

Figures

Figure S1. Preparative HPLC chromatograms for the purified compounds of soybean leaves. 14

Figure S2. Mass spectra of precursors and fragments of purified compounds from CJ........................ 15

Figure S3. Mass spectra of precursors and fragments of purified compounds from DW. 16

Figure S4. Mass spectra of precursors and fragments of purified compounds from NP....................... 17

Figure S5. Mass spectra of precursors and fragments of purified compounds from WR. 19

Figure S6. Spectral purity calculation for the purified compounds CJ1... 20

Figure S7. Spectral purity calculation for the purified compounds CJ2... 21

Figure S8. Spectral purity calculation for the purified compounds DW1. 22

Figure S9. Spectral purity calculation for the purified compounds DW2. 23

Figure S10. Spectral purity calculation for the purified compounds NP1.. 24

Figure S11. Spectral purity calculation for the purified compounds NP2.. 25

Figure S12. Spectral purity calculation for the purified compounds WR1. 26

Figure S13. Spectral purity calculation for the purified compounds WR2. 27

Figure S14. Spectral purity calculation for the purified compounds WR3. 28

Figure S15. Overlaid single ion recoding mass chromatograms of 31 compounds. 29

Figure S16. Multivariate analysis of SL metabolites. 30

Figure S17. Prediction results of Neural model of machine learning. 31

Script

Script S1. Python code for prediction of the discriminative classes. 32

Table S1. Purity of compounds used in method validation and conditions of mass detection

No.[a]	Class [b]	Compound		Purity (fraction)		Mass detection				
		Name	Source [c]	Stand-alone [d]	Calculated [e]	*m/z* [f]	Polarity [g]	RT [h]	SIR-S [i]	SIR-E [j]
1	FL	quercetin 3-*O*-tri-glycoside A	CJ1	0.950	1.235	771.20	N	1.04	0.80	1.41
2	FL	quercetin 3-*O*-tri-glycoside B	CJ2	0.640	0.640	771.20	N	1.09	0.80	1.41
3	FL	quercetin 3-*O*-di-glycoside A	NP1	0.950	1.026	625.20	N	1.34	1.00	1.73
4	FL	quercetin 3-*O*-di-glycoside B	NP2	0.900	0.900	625.20	N	1.40	1.00	1.73
5	IS	daidzin	reagent	0.980	1.011	417.11	P	1.45	1.10	1.75
6	FL	kaempferol 3-*O*-tri-glycoside A	DW1	0.988	1.086	755.20	N	1.48	1.18	1.95
7	FL	kaempferol 3-*O*-tri-glycoside B	DW2	0.900	0.900	755.20	N	1.62	1.18	1.95
8	IS	glycitin	reagent	0.980	0.984	447.12	P	1.67	1.40	1.97
9	FL	kaempferol 3-*O*-tri-glycoside C	WR1	0.900	0.900	739.20	N	2.01	1.80	2.51
10	FL	rutin	reagent	0.940	0.940	609.15	N	2.45	1.90	2.69
11	FL	isoquercitrin	reagent	0.900	0.900	463.10	N	2.61	2.30	2.81
12	IS	genistin	reagent	0.980	0.985	433.11	P	2.70	2.34	2.91
13	FL	kaempferol 3-*O*-di-glycoside A	WR2	0.960	0.960	593.15	N	2.72	2.38	3.15
14	FL	kaempferol 3-*O*-di-glycoside B	WR3	0.977	0.977	593.15	N	2.91	2.38	3.15
15	IS	malonyl daidzin	reagent	0.900	0.912	503.11	P	2.92	2.68	3.13
16	IS	malonyl glycitin	reagent	0.900	0.901	533.12	P	2.98	2.74	3.21
17	FL	astragalin	reagent	0.970	0.970	447.09	N	3.03	2.80	3.29
18	FE	apigenin 7-*O*-glucoside	reagent	0.970	0.970	431.02	N	3.16	3.02	3.31
19	IS	acetyl daidzin	reagent	0.900	0.900	459.12	P	3.31	3.04	3.55
20	IS	acetyl glycitin	reagent	0.900	0.900	489.13	P	3.38	3.14	3.61
21	IS	malonyl genistin	reagent	0.900	0.900	519.11	P	3.45	3.24	3.71
22	IS	daidzein	reagent	0.980	0.980	255.06	P	3.79	3.60	4.01
23	IS	acetyl genistin	reagent	0.900	0.900	475.12	P	3.97	3.70	4.09
24	IS	glycitein	reagent	0.980	0.980	285.07	P	4.01	3.78	4.11
25	FE	luteolin	reagent	0.980	0.980	285.04	N	4.19	3.92	4.35
26	FL	quercetin	reagent	0.950	0.950	301.04	N	4.21	3.94	4.37
27	IS	genistein	reagent	0.980	0.980	271.05	P	5.40	5.14	5.71
28	FE	apigenin	reagent	0.950	0.950	269.05	N	5.51	5.18	5.75
29	CM	coumestrol	reagent	0.950	0.950	267.04	N	5.58	5.24	5.85
30	FL	kaempferol	reagent	0.970	0.970	285.05	N	5.65	5.40	6.21
31	FL	isorhamnetin	reagent	0.950	0.950	315.06	N	5.71	5.50	6.31

[a] Numbered in the order of retention time (RT). [b] CM, coumestrol; FE, flavone; FL, flavonol; and IS, isoflavone. [c] CJ1–2, from CJ (Cheng-ja soybean variety); DW1–2, from DW (Dae-won); NP1–2, from NP (Nok-poong); and WR1–3, WR(Woo-ram). [d] Purity of the single compound itself. [e] Purity in the combined solution. [f] Mass-to-charge ratio. [g] N, negative and P, positive. [h] Average RT (min) obtained from the validation test. [i] Single ion recoding start (min). [j] Single ion recoding end (min).

Table S2. Content of phenolic classes in the soybean leaves by cultivation days

(A) per dry weight of soybean leaf (unit: µg/g dry weight)

		Compound class [b]			
Day	Variety [a]	CM	FE	FL	IS
30	CJ (PJ)	LL [c]	14.85 ± 1.96a	1331.83 ± 132.09a	89.43 ± 2.52b
	CJ (YC)	LL	13.20 ± 2.24a	1640.15 ± 234.84a	395.63 ± 56.41a
	DW	5.43 ± 2.47	6.83 ± 1.12b	482.38 ± 93.76b	249.30 ± 59.96a
60	CJ (PJ)	LL	30.44 ± 6.24a	1670.39 ± 202.61a	481.58 ± 137.4a
	CJ (YC)	LL	18.40 ± 4.39a	1619.26 ± 236.13a	327.26 ± 105.64a
	DW	LL	4.40 ± 0.31b	469.62 ± 165.7b	186.34 ± 91.33a
90	CJ (PJ)	0.38 ± 0.00a	14.66 ± 2.39a	1608.27 ± 165.18a	1082.93 ± 69.5a
	CJ (YC)	1.08 ± 0.48a	3.21 ± 0.93b	1632.72 ± 177.75a	974.55 ± 220.84a
	DW	9.37 ± 4.51a	0.99 ± 0.00ab	210.58 ± 23.1b	213.10 ± 42.63b
120	CJ (PJ)	3.96 ± 2.07b	15.77 ± 2.85a	1833.89 ± 310.29a	502.60 ± 92.12b
	CJ (YC)	6.64 ± 2.29b	27.31 ± 11.47a	2010.03 ± 267.83a	869.04 ± 164.96a
	DW	32.48 ± 12.41a	8.06 ± 3.22a	854.97 ± 294.55b	279.24 ± 29.72b

(B) per plant (unit: µg/plant)

		Compound class [b]			
Day	Variety [a]	CM	FE	FL	IS
30	CJ (PJ)	LL	26.74 ± 3.52a	2397.30 ± 237.76ab	160.97 ± 4.54b
	CJ (YC)	LL	23.36 ± 3.97a	2903.07 ± 415.67a	700.27 ± 99.84a
	DW	17.61 ± 8.01	22.15 ± 3.63a	1564.84 ± 304.17b	808.71 ± 194.5a
60	CJ (PJ)	LL	499.47 ± 102.37a	27407.77 ± 3324.48a	7901.78 ± 2254.47a
	CJ (YC)	LL	195.28 ± 46.55b	17186.79 ± 2506.25b	3473.51 ± 1121.28ab
	DW	LL	54.30 ± 3.81b	5798.90 ± 2046.11c	2300.97 ± 1127.74b
90	CJ (PJ)	7.42 ± 0.00a	289.10 ± 47.16a	31718.30 ± 3257.67b	21357.47 ± 1370.64a
	CJ (YC)	31.44 ± 13.92a	93.73 ± 26.99b	47633.08 ± 5185.82a	28431.46 ± 6442.86a
	DW	315.29 ± 151.65a	33.26 ± 0.00b	7083.83 ± 777.16c	7168.81 ± 1434.15b
120	CJ (PJ)	123.91 ± 64.81b	492.98 ± 89.26a	57338.33 ± 9701.55a	15714.14 ± 2880.15ab
	CJ (YC)	170.98 ± 59.07b	703.32 ± 295.49a	51766.33 ± 6897.66a	22381.13 ± 4248.35a
	DW	810.4 ± 309.57a	201.14 ± 80.21a	21329.34 ± 7348.19b	6966.22 ± 741.34b

[a] CJ, Cheng-ja soybean variety; DW, Dae-won soybean variety; PJ, Pa-ju city; and YC, Yeon-chen city. [b] CM, coumestrol; FE, flavone; FL, flavonol; and IS, isoflavone. [c] Lower than limit of quantification. Statistical letter after the numbers was compared within the same cultivation day and compound class using Student's *t*-test for each pair. ($p < 0.05$)

Table S3. Content of phenolic compounds in soybean leaves per dry weight.
(A) day 30 and 60 and (B) day 90 and 120 (unit: µg/g dry weight).

(A)		Day	30			60		
No.	Class [a]	Compound	CJ (PJ) [b]	CJ (YC)	DW (PJ)	CJ (PJ)	CJ (YC)	DW (PJ)
1	FL	quercetin 3-*O*-tri-glycoside A	139.01 ± 10.85ab	310.38 ± 162.52a	2.51 ± 1.46b	638.73 ± 0.00b	749.12 ± 0.00a	3.63 ± 1.52c
2	FL	quercetin 3-*O*-tri-glycoside B	45.22 ± 4.38b	299.21 ± 80.72a	0.6 ± 0.00b	256.79 ± 0.00ab	431.06 ± 57.35a	0.56 ± 0.26b
3	FL	quercetin 3-*O*-di-glycoside A	20.96 ± 3.29b	61.32 ± 14.18a	LL [c]	438.72 ± 99.50a	232.42 ± 57.25a	LL
4	FL	quercetin 3-*O*-di-glycoside B	13.76 ± 1.60a	59.9 ± 14.86b	LL	311.63 ± 68.08a	183.61 ± 43.52a	LL
5	IS	daidzin	LL	16.9 ± 3.61a	9.18 ± 3.25a	20.24 ± 9.30a	12.64 ± 7.00a	15.21 ± 9.22a
6	FL	kaempferol 3-*O*-tri-glycoside A	784.13 ± 28.24a	241.08 ± 96.88b	LL	642.2 ± 0.00a	626.46 ± 42.70a	411.65 ± 0.00a
7	FL	kaempferol 3-*O*-tri-glycoside B	LL	548.7 ± 73.39	LL	LL	LL	380.86 ± 0.00
8	IS	glycitin	LL	LL	LL	LL	LL	LL
9	FL	kaempferol 3-*O*-tri-glycoside C	314.43 ± 12.58ab	187.65 ± 21.44b	436.99 ± 192.84a	444.18 ± 0.00a	365.73 ± 68.57a	330.71 ± 96.63a
10	FL	rutin	10.49 ± 0.95b	65.38 ± 12.99a	LL	238.79 ± 55.65a	146.91 ± 38.76ab	0.69 ± 0.07b
11	FL	isoquercitrin	4.42 ± 0.48b	14.86 ± 3.22a	LL	42.62 ± 10.77a	25.37 ± 6.34a	LL
12	IS	genistin	11.02 ± 0.46c	58.23 ± 8.91a	34.91 ± 8.36b	32.4 ± 11a	26.24 ± 8.35a	26.73 ± 11.93a
13	FL	kaempferol 3-*O*-di-glycoside A	92.64 ± 2.76b	47.21 ± 5.19b	167.82 ± 29.65a	158.47 ± 26.28a	100.92 ± 25.26a	114.69 ± 22.66a
14	FL	kaempferol 3-*O*-di-glycoside B	52.75 ± 3.17b	29.26 ± 3.89b	125.44 ± 25.34a	73.28 ± 13.25a	57.77 ± 17.02a	60.12 ± 13.69a
15	IS	malonyl daidzin	3.3 ± 0.30b	66.62 ± 12.34a	49.09 ± 14.81a	73.92 ± 33.10a	60.93 ± 29.36a	49.27 ± 28.12a
16	IS	malonyl glycitin	LL	2.21 ± 0.42a	1.11 ± 0.25a	13.59 ± 3.54a	11.81 ± 2.60a	3.43 ± 0.48a
17	FL	astragalin	10.86 ± 0.69ab	5.3 ± 1.82b	12.7 ± 2.47a	13.14 ± 0.98a	8.55 ± 1.92a	1.22 ± 0.29b
18	FE	apigenin 7-*O*-glucoside	LL	LL	LL	LL	LL	LL
19	IS	acetyl daidzin	LL	LL	LL	1.87 ± 0.35a	2.39 ± 1.13a	3.54 ± 0.80a
20	IS	acetyl glycitin	LL	LL	LL	LL	LL	LL
21	IS	malonyl genistin	23.55 ± 1.84c	239.18 ± 30.40a	119.89 ± 32.16b	256.97 ± 83.09a	156.4 ± 62.51a	89.9 ± 46.41a
22	IS	daidzein	31.35 ± 0.81a	9.38 ± 1.30c	18.15 ± 3.02b	67.5 ± 15.51a	35.75 ± 10.03ab	11.93 ± 2.41b
23	IS	acetyl genistin	LL	LL	LL	0.76 ± 0.00	LL	LL
24	IS	glycitein	4.88 ± 0.29a	LL	2.81 ± 0.00b	9.05 ± 2.54a	14.56 ± 4.69a	1.81 ± 0.00a
25	FE	luteolin	14.85 ± 1.96a	13.2 ± 2.24a	LL	30.22 ± 6.42a	18.4 ± 4.39a	LL
26	FL	quercetin	LL	LL	LL	LL	LL	LL
27	IS	genistein	15.34 ± 1.37a	6.21 ± 0.36a	20.78 ± 8.47a	13.37 ± 2.36a	16.05 ± 4.29a	3.99 ± 0.42a
28	FE	apigenin	LL	LL	6.83 ± 1.12	1.08 ± 0.00b	LL	4.4 ± 0.31a
29	CM	coumestrol	LL	LL	5.43 ± 2.47	LL	LL	LL
30	FL	kaempferol	LL	LL	LL	LL	LL	LL
31	FL	isorhamnetin	LL	LL	LL	LL	LL	LL

to be continued.

(B)		Day	90			120		
No.	Class	Compound	CJ (PJ)	CJ (YC)	DW (PJ)	CJ (PJ)	CJ (YC)	DW (PJ)
1	FL	quercetin 3-*O*-tri-glycoside A	LL	544.88 ± 0.00a	3.72 ± 0.92b	213.09 ± 0.00b	238.81 ± 0.00a	2.8 ± 0.60c
2	FL	quercetin 3-*O*-tri-glycoside B	LL	336.46 ± 110.44a	0.64 ± 0.33b	52.94 ± 0.00a	354.38 ± 94.96a	1.19 ± 0.69a
3	FL	quercetin 3-*O*-di-glycoside A	537.34 ± 56.03a	187.94 ± 32.85b	LL	492.1 ± 125.31a	313.32 ± 118.90a	LL
4	FL	quercetin 3-*O*-di-glycoside B	391.89 ± 45.99a	236.67 ± 57.40a	LL	441.13 ± 113.13a	257.24 ± 86.06a	LL
5	IS	daidzin	91.7 ± 6.47a	102.83 ± 22.80a	6.12 ± 2.46b	56.79 ± 14.26a	88.94 ± 34.19a	17.43 ± 2.90a
6	FL	kaempferol 3-*O*-tri-glycoside A	LL	568.45 ± 254.08	LL	90.74 ± 0.00a	547.49 ± 144.14a	483.92 ± 152.07a
7	FL	kaempferol 3-*O*-tri-glycoside B	LL	557.28 ± 0.00	LL	58.07 ± 0.00a	113.66 ± 0.00a	281.32 ± 120.57a
8	IS	glycitin	7.34 ± 0.96a	1.88 ± 0.00a	LL	30.18 ± 9.97a	16.06 ± 6.74a	6.42 ± 1.29a
9	FL	kaempferol 3-*O*-tri-glycoside C	LL	362.85 ± 65.85	LL	452.79 ± 206.64a	274.19 ± 71.94a	158.67 ± 62.82a
10	FL	rutin	345.33 ± 32.57a	140.16 ± 26.96b	LL	272.77 ± 69.71a	166.29 ± 47.74a	0.94 ± 0.00a
11	FL	isoquercitrin	87.56 ± 11.22a	29.42 ± 6.20b	LL	114.14 ± 19.18a	73.7 ± 32.32a	LL
12	IS	genistin	80.48 ± 4.50a	75.15 ± 15.71a	21.56 ± 3.63b	54.46 ± 5.83a	130.11 ± 51.06a	20.93 ± 10.86a
13	FL	kaempferol 3-*O*-di-glycoside A	170.76 ± 12.59a	58.03 ± 10.14b	136.31 ± 12.54a	95.84 ± 21.96b	49.62 ± 13.38b	173 ± 27.30a
14	FL	kaempferol 3-*O*-di-glycoside B	63.72 ± 8.02a	31.81 ± 5.52b	67.77 ± 8.56a	48.87 ± 11.26ab	32.02 ± 7.56b	90.43 ± 33.00a
15	IS	malonyl daidzin	295.22 ± 26.46b	454.11 ± 92.36a	48.33 ± 12.76c	47.53 ± 14.13a	153.86 ± 62.11a	35.87 ± 16.19a
16	IS	malonyl glycitin	61.27 ± 4.15a	31.68 ± 7.93b	6.19 ± 1.07c	33.59 ± 7.23a	53.67 ± 19.34a	10.76 ± 0.71a
17	FL	astragalin	11.66 ± 2.69a	5.72 ± 3.12ab	3.16 ± 1.76b	14.32 ± 2.02a	7.92 ± 3.45a	20.4 ± 12.10a
18	FE	apigenin 7-*O*-glucoside	LL	LL	LL	LL	LL	6.38 ± 4.74
19	IS	acetyl daidzin	2.89 ± 0.33a	3.68 ± 0.79a	1.4 ± 0.22a	LL	1.28 ± 0.45a	2.84 ± 0.34a
20	IS	acetyl glycitin	0.2 ± 0.00	0.22 ± 0.00	LL	LL	LL	LL
21	IS	malonyl genistin	453.88 ± 24.18a	470.99 ± 83.37a	123.18 ± 21.77b	61.92 ± 15.45a	262.75 ± 146.86a	57.68 ± 26.59a
22	IS	daidzein	62.72 ± 3.2a	19.12 ± 3.98b	6.55 ± 1.19c	130.12 ± 11.08a	152.15 ± 53.04a	103.04 ± 21.90a
23	IS	acetyl genistin	1.71 ± 0.59a	1.16 ± 0.37a	LL	LL	LL	LL
24	IS	glycitein	13 ± 0.75a	1.28 ± 0.00b	LL	79.87 ± 19.81a	36.67 ± 12.89ab	27.61 ± 4.32b
25	FE	luteolin	14.66 ± 2.39a	3.21 ± 0.93b	LL	15.77 ± 2.85a	26.32 ± 10.88a	LL
26	FL	quercetin	LL	LL	LL	19.16 ± 15.74a	73.66 ± 39.59a	4.58 ± 1.17a
27	IS	genistein	12.7 ± 1.06a	3.17 ± 0.00b	3.13 ± 0.00b	43.14 ± 2.23a	84.37 ± 31.15a	44.01 ± 7.99a
28	FE	apigenin	LL	LL	0.99 ± 0.00	LL	2.47 ± 0.16a	4.87 ± 0.94a
29	CM	coumestrol	0.38 ± 0.00a	1.08 ± 0.48a	9.37 ± 4.51a	3.96 ± 2.07b	6.64 ± 2.29b	32.48 ± 12.41a
30	FL	kaempferol	LL	LL	LL	22.45 ± 0.00a	120.32 ± 53.74a	219.53 ± 54.79a
31	FL	isorhamnetin	LL	LL	LL	10.64 ± 0.00	15.11 ± 5.39	LL

[a] CM, coumestrol; FE, flavones; FL, flavonols; and IS, isoflavones. [b] CJ, Cheng-ja soybean variety; DW, Dae-won soybean variety; PJ, Pa-ju city; and YC, Yeon-chen city. [c] Lower than limit of quantification. Data are shown as the mean ± standard error of means (n = 1~5). Different letters in the same row of same compound and day indicate significant differences using comparisons of each pair by Student's *t*-test ($p < 0.05$).

Table S4**.** Content of phenolic compounds in soybean leaves per plant.
(A) day 30 and 60 and (B) day 90 and 120 (unit: µg/plant)

(A)		Day	30			60		
No.	Class [a]	Compound	CJ (PJ) [b]	CJ (YC)	DW (PJ)	CJ (PJ)	CJ (YC)	DW (PJ)
1	FL	quercetin 3-*O*-tri-glycoside A	250 ± 20ab	549 ± 288a	8 ± 5b	10480 ± 0.00a	7951 ± 0.00b	45 ± 19c
2	FL	quercetin 3-*O*-tri-glycoside B	81 ± 8b	530 ± 143a	2 ± 0.00b	4213 ± 0.00a	4575 ± 609a	7 ± 3b
3	FL	quercetin 3-*O*-di-glycoside A	38 ± 6b	109 ± 25a	LL	7198 ± 1633a	2467 ± 608b	LL
4	FL	quercetin 3-*O*-di-glycoside B	25 ± 3b	106 ± 26a	LL	5113 ± 1117a	1949 ± 462b	LL
5	IS	daidzin	LL	30 ± 6a	30 ± 11a	332 ± 153a	134 ± 74a	188 ± 114a
6	FL	kaempferol 3-*O*-tri-glycoside A	1411 ± 51a	427 ± 171b	LL	10537 ± 0.00a	6649 ± 453a	5083 ± 0.00a
7	FL	kaempferol 3-*O*-tri-glycoside B	LL	971 ± 130	LL	LL	LL	4703 ± 0.00
8	IS	glycitin	LL	LL	LL	LL	LL	LL
9	FL	kaempferol 3-*O*-tri-glycoside C	566 ± 23b	332 ± 38b	1418 ± 626a	7288 ± 0.00a	3882 ± 728a	4084 ± 1193a
10	FL	rutin	19 ± 2b	116 ± 23a	LL	3918 ± 913a	1559 ± 411b	9 ± 1b
11	FL	isoquercitrin	8 ± 1b	26 ± 6a	LL	699 ± 177a	269 ± 67a	LL
12	IS	genistin	20 ± 1b	103 ± 16a	113 ± 27a	532 ± 181a	279 ± 89a	330 ± 147a
13	FL	kaempferol 3-*O*-di-glycoside A	167 ± 5b	84 ± 9b	544 ± 96a	2600 ± 431a	1071 ± 268b	1416 ± 280b
14	FL	kaempferol 3-*O*-di-glycoside B	95 ± 6b	52 ± 7b	407 ± 82a	1202 ± 217a	613 ± 181b	742 ± 169ab
15	IS	malonyl daidzin	6 ± 1b	118 ± 22a	159 ± 48a	1213 ± 543a	647 ± 312a	608 ± 347a
16	IS	malonyl glycitin	LL	4 ± 1a	4 ± 1a	223 ± 58a	125 ± 28a	42 ± 6a
17	FL	astragalin	20 ± 1b	9 ± 3b	41 ± 8a	216 ± 16a	91 ± 20b	15 ± 4c
18	FE	apigenin 7-*O*-glucoside	LL	LL	LL	LL	LL	LL
19	IS	acetyl daidzin	LL	LL	LL	31 ± 6a	25 ± 12a	44 ± 10a
20	IS	acetyl glycitin	LL	LL	LL	LL	LL	LL
21	IS	malonyl genistin	42 ± 3b	423 ± 54a	389 ± 104a	4216 ± 1363a	1660 ± 664ab	1110 ± 573b
22	IS	daidzein	56 ± 1a	17 ± 2b	59 ± 10a	1107 ± 254a	379 ± 106b	147 ± 30b
23	IS	acetyl genistin	LL	LL	LL	13 ± 0.00	LL	LL
24	IS	glycitein	9 ± 1a	LL	9 ± 0.00a	148 ± 42a	155 ± 50a	22 ± 0.00a
25	FE	luteolin	27 ± 4a	23 ± 4a	LL	496 ± 105a	195 ± 47b	LL
26	FL	quercetin	LL	LL	LL	LL	LL	LL
27	IS	genistein	28 ± 2a	11 ± 1a	67 ± 27a	219 ± 39a	170 ± 46ab	49 ± 5b
28	FE	apigenin	LL	LL	22 ± 4	18 ± 0.00b	LL	54 ± 4a
29	CM	coumestrol	LL	LL	18 ± 8	LL	LL	LL
30	FL	kaempferol	LL	LL	LL	LL	LL	LL
31	FL	isorhamnetin	LL	LL	LL	LL	LL	LL

to be continued.

(B)		Day	90			120		
No.	Class	Compound	CJ (PJ)	CJ (YC)	DW (PJ)	CJ (PJ)	CJ (YC)	DW (PJ)
1	FL	quercetin 3-*O*-tri-glycoside A	LL	15896 ± 0.00a	125 ± 31b	6663 ± 0.00a	6150 ± 0.00b	70 ± 15c
2	FL	quercetin 3-*O*-tri-glycoside B	LL	9816 ± 3222a	21 ± 11b	1655 ± 0.00a	9127 ± 2446a	30 ± 17a
3	FL	quercetin 3-*O*-di-glycoside A	10597 ± 1105a	5483 ± 958b	LL	15386 ± 3918a	8069 ± 3062a	LL
4	FL	quercetin 3-*O*-di-glycoside B	7729 ± 907a	6905 ± 1675a	LL	13792 ± 3537a	6625 ± 2216a	LL
5	IS	daidzin	1808 ± 128a	3000 ± 665a	206 ± 83b	1775 ± 446a	2290 ± 880a	435 ± 72a
6	FL	kaempferol 3-*O*-tri-glycoside A	LL	16584 ± 7413	LL	2837 ± 0.00a	14100 ± 3712a	12073 ± 3794a
7	FL	kaempferol 3-*O*-tri-glycoside B	LL	16258 ± 0.00	LL	1816 ± 0.00a	2927 ± 0.00a	7018 ± 3008a
8	IS	glycitin	145 ± 19a	55 ± 0.00a	LL	944 ± 312a	414 ± 174a	160 ± 32a
9	FL	kaempferol 3-*O*-tri-glycoside C	LL	10586 ± 1921	LL	14157 ± 6461a	7061 ± 1853a	3958 ± 1567a
10	FL	rutin	6811 ± 642a	4089 ± 786b	LL	8528 ± 2179a	4283 ± 1230a	23 ± 0.00a
11	FL	isoquercitrin	1727 ± 221a	858 ± 181b	LL	3569 ± 600a	1898 ± 832a	LL
12	IS	genistin	1587 ± 89a	2193 ± 458a	725 ± 122b	1703 ± 182a	3351 ± 1315a	522 ± 271a
13	FL	kaempferol 3-*O*-di-glycoside A	3368 ± 248b	1693 ± 296c	4585 ± 422a	2996 ± 687ab	1278 ± 344b	4316 ± 681a
14	FL	kaempferol 3-*O*-di-glycoside B	1257 ± 158b	928 ± 161b	2280 ± 288a	1528 ± 352a	825 ± 195a	2256 ± 823a
15	IS	malonyl daidzin	5822 ± 522b	13248 ± 2694a	1626 ± 429c	1486 ± 442a	3962 ± 1599a	895 ± 404a
16	IS	malonyl glycitin	1208 ± 82a	924 ± 231a	208 ± 36b	1050 ± 226a	1382 ± 498a	268 ± 18a
17	FL	astragalin	230 ± 53a	167 ± 91a	106 ± 59a	448 ± 63a	204 ± 89a	509 ± 302a
18	FE	apigenin 7-*O*-glucoside	LL	LL	LL	LL	LL	159 ± 118
19	IS	acetyl daidzin	57 ± 6a	107 ± 23a	47 ± 7a	LL	33 ± 12a	71 ± 8a
20	IS	acetyl glycitin	4 ± 0.00	6 ± 0.00	LL	LL	LL	LL
21	IS	malonyl genistin	8951 ± 477b	13741 ± 2432a	4144 ± 732c	1936 ± 483a	6767 ± 3782a	1439 ± 663a
22	IS	daidzein	1237 ± 63a	558 ± 116b	220 ± 40c	4068 ± 346a	3918 ± 1366a	2571 ± 546a
23	IS	acetyl genistin	34 ± 12a	34 ± 11a	LL	LL	LL	LL
24	IS	glycitein	256 ± 15a	37 ± 0.00b	LL	2497 ± 619a	944 ± 332b	689 ± 108b
25	FE	luteolin	289 ± 47a	94 ± 27b	LL	493 ± 89a	678 ± 280a	LL
26	FL	quercetin	LL	LL	LL	599 ± 492a	1897 ± 1020a	114 ± 29a
27	IS	genistein	250 ± 21a	92 ± 0.00b	105 ± 0.00b	1349 ± 70a	2173 ± 802a	1098 ± 199a
28	FE	apigenin	LL	LL	33 ± 0.00	LL	64 ± 4a	122 ± 24a
29	CM	coumestrol	7 ± 0.00a	31 ± 14a	315 ± 152a	124 ± 65b	171 ± 59b	810 ± 310a
30	FL	kaempferol	LL	LL	LL	702 ± 0.00a	3099 ± 1384a	5477 ± 1367a
31	FL	isorhamnetin	LL	LL	LL	333 ± 0.00	389 ± 139	LL

[a] CM, coumestrol; FE, flavones; FL, flavonols; and IS, isoflavones. [b] CJ, Cheng-ja soybean variety; DW, Dae-won soybean variety; PJ, Pa-ju city; and YC, Yeon-chen city. [c] Lower than limit of quantification. Data are shown as the mean ± standard error of means (n = 1~5). Different letters in the same row of same compound and day indicate significant differences using comparisons of each pair by Student's *t*-test ($p < 0.05$).

Table S5. Summary of machine learning models.

(A) Summary of established models using SIMCA

Model [a]	Comparison class of the dataset	Number of components (NC)	Number of observation in the model	R^2X(cum) [b]	R^2Y(cum) [c]	Q^2(cum) [d]
OPLS	All	1+6+0	57	0.812	0.783	0.638
OPLS-DA	Variety	1+2+0	56	0.602	0.798	0.729
	City	1+1+0	39	0.664	0.449	0.406

[a] OPLS, orthogonal partial-least squares; OPLS-DA; orthogonal partial-least squares discriminant analysis. [b] Cumulative X-variation modeled after NC. [c] Cumulative Y-variation modeled after NC (OPLS). [d] Cumulative overall cross-validated R^2X for a PC model; R^2Y for a OPLS model.

(B) Misclassification table of OPLS-DA to discriminate variety

	Members	Correct	CJ	DW	No class (Y_{Pred} ≦ 0)
CJ [a]	39	100.00%	39	0	0
DW [b]	19	100.00%	0	19	0
No class	0		0	0	0
Total	58	100.00%	39	19	0
Fisher's probability	1.10×10^{-15}				

[a] CJ: Cheng-ja soybean variety. *[b]* DW: Dae-won soybean variety.

(C) Misclassification table of OPLS-DA to discriminate city

	Members	Correct	YC	PJ	No class (Y_{Pred} ≦ 0)
YC [a]	19	84.21%	16	3	0
PJ [b]	20	85.00%	3	17	0
No class	0		0	0	0
Total	39	84.62%	19	20	0
Fisher's probability	1.70×10^{-5}				

[a] PJ: Pa-ju city. [b] YC: Yeon-chen city.

(D) Summary of machine learning models using JMP pro

Model [a]	Class of dataset	Number of layers or trees	Generalized R^2 [b]			Misclassificaiton rate		
			traning	validation	test	traning	validation	test
NU	variety	3+10	0.981	0.950	0.936	0	0	0
	day	3+6	0.999	0.986	0.960	0	0	0.09
	city	3+2	0.999	0.998	0.991	0	0	0
BF	day	5	0.809	0.537	0.806	0.09	0.17	0.18
BT	variety	50	0.999	0.999	0.999	0	0	0
	city	50	0.989	0.943	NA	0	0	NA

[a] NU, neural; BF, bootstrap forest; and BT, boosted tree. [b] Generalized R^2 measure simplifies to the traditional R^2 for continuous normal responses in the standard least squares setting. Generalized R^2 is also known as the Nagelkerke or Craig and Uhler R^2, which is a normalized version of Cox and Snell's pseudo R^2.

Table S6. Key compounds obtained using cut-off criteria of S-plot (SIMCA)

Model [a]	Class of discrimination	Compound	p[1] [b]	$p_{(corr)}[1]$ [c]
OPLS	Day	daidzein	0.345	0.636
		genistein	0.203	0.512
		glycitein	0.217	0.696
		glycitin	0.110	0.592
		malonyl glycitin	0.174	0.512
OPLS-DA	Variety	apigenin	0.049	0.656
		isoquercitrin	−0.200	−0.661
		kaempferol 3-*O*-di-glycoside B	0.145	0.501
		luteolin	−0.095	−0.556
		malonyl glycitin	−0.114	−0.506
		quercetin 3-*O*-di-glycoside A	−0.500	−0.688
		quercetin 3-*O*-di-glycoside B	−0.457	−0.705
		rutin	−0.386	−0.703
OPLS-DA	City	astragalin	0.060	0.596
		kaempferol 3-*O*-di-glycoside A	0.221	0.633
		kaempferol 3-*O*-di-glycoside B	0.161	0.699
		malonyl daidzin	−0.290	−0.519
		quercetin 3-*O*-tri-glycoside A	−0.340	−0.597
		quercetin 3-*O*-tri-glycoside B	−0.448	−0.705

[a] OPLS, orthogonal partial-least squares; and OPLS-DA, orthogonal partial-least squares discriminant analysis. [b] Modeled covariation. [c] Correlation coefficient.

Table S7. Key compounds obtained using cut-off criteria of VIP (SIMCA)

Model [a]	Class of discrimination	Compound	VIP [b]
OPLS	Day	quercetin 3-*O*-di-glycoside A	2.08
		quercetin 3-*O*-di-glycoside B	1.94
		rutin	1.57
		kaempferol 3-*O*-tri-glycoside A	1.52
		daidzein	1.51
		malonyl daidzin	1.35
		quercetin 3-*O*-tri-glycoside A	1.31
		kaempferol 3-*O*-tri-glycoside C	1.25
		malonyl genistin	1.25
		daidzin	1.01
		quercetin 3-*O*-tri-glycoside B	1.00
		kaempferol	0.97
		isoquercitrin	0.95
		glycitein	0.93
		genistein	0.93
		kaempferol 3-*O*-tri-glycoside B	0.81
OPLS-DA	Variety	quercetin 3-*O*-di-glycoside A	2.31
		quercetin 3-*O*-di-glycoside B	2.07
		rutin	1.77
		kaempferol 3-*O*-tri-glycoside A	1.54
		malonyl genistin	1.20
		quercetin 3-*O*-tri-glycoside B	1.15
		malonyl daidzin	1.08
		kaempferol 3-*O*-tri-glycoside C	1.04
		quercetin 3-*O*-tri-glycoside A	1.02
		isoquercitrin	0.92
		kaempferol 3-*O*-di-glycoside A	0.81
OPLS-DA	City	quercetin 3-*O*-tri-glycoside B	1.88
		quercetin 3-*O*-di-glycoside A	1.82
		kaempferol 3-*O*-tri-glycoside C	1.74
		quercetin 3-*O*-di-glycoside B	1.56
		malonyl genistin	1.56
		malonyl daidzin	1.41
		quercetin 3-*O*-tri-glycoside A	1.37
		rutin	1.31
		kaempferol 3-*O*-tri-glycoside A	1.10
		kaempferol 3-*O*-di-glycoside A	0.94

[a] OPLS, orthogonal partial-least squares; and OPLS-DA, orthogonal partial-least squares discriminant analysis. [b] Variable importance projection.

Table S8. Key compounds obtained using cut-off criteria of machine learning (JMP pro).

Model [a]	Class of discrimination	Compound	Number of splits	G^2 [b]	Portion [c]
BF	Day	glycitein	3	7.96	0.22
		malonyl glycitin	2	6.05	0.17
		daidzein	2	5.00	0.14
		genistein	1	4.82	0.13
		acetyl daidzin	2	2.90	0.08
		quercetin	2	2.56	0.07
		luteolin	1	2.49	0.07
		astragalin	1	2.13	0.06
BT	Variety	luteolin	22	959.18	0.44
		quercetin 3-*O*-tri-glycoside A	26	593.46	0.27
		apigenin	28	581.90	0.27
BT	City	acetyl daidzin	22	271.97	0.12
		kaempferol 3-*O*-tri-glycoside A	12	269.72	0.12
		kaempferol 3-*O*-di-glycoside A	14	258.71	0.12
		kaempferol 3-*O*-tri-glycoside C	11	235.14	0.11
		glycitein	11	216.02	0.10
		quercetin 3-*O*-tri-glycoside B	12	183.69	0.08
		malonyl genistin	9	163.67	0.08
		luteolin	8	160.02	0.07
		genistein	7	154.98	0.07
		quercetin 3-*O*-di-glycoside B	6	105.74	0.05

[a] BF, bootstrap forest; and BT, boosted tree. [b] Likelihood ratio chi-square. [c] The portion is given among the compounds which have G^2 value according to the result of machine learning modeling.

Table S9. Example of prediction results executed by Python code.

No.	Day			Variety			City		
	original	pred. Neural[a]	pred. BF[b]	original	pred. Neural	pred. BT[c]	original	pred. Neural	pred. BT
0	30	30	30	CJ	CJ	CJ	YC	YC	YC
1	30	30	60*	CJ	CJ	CJ	YC	YC	YC
2	90	90	90	CJ	CJ	CJ	YC	YC	YC
3	30	60*	30	DW	DW	DW	PJ	PJ	PJ
4	90	90	60*	DW	DW	DW	PJ	PJ	PJ
5	30	30	30	CJ	CJ	CJ	PJ	PJ	PJ
6	30	30	30	CJ	CJ	CJ	PJ	PJ	PJ
7	30	30	30	CJ	CJ	CJ	PJ	PJ	PJ
8	60	60	60	CJ	CJ	CJ	PJ	PJ	PJ
9	60	60	60	CJ	CJ	CJ	PJ	PJ	PJ
10	120	120	120	CJ	CJ	CJ	PJ	PJ	PJ

[a] Prediction of neural model, [b] Prediction of bootstrap forest model, [c] Prediction of boosted tree model.
* Misclassified prediction results

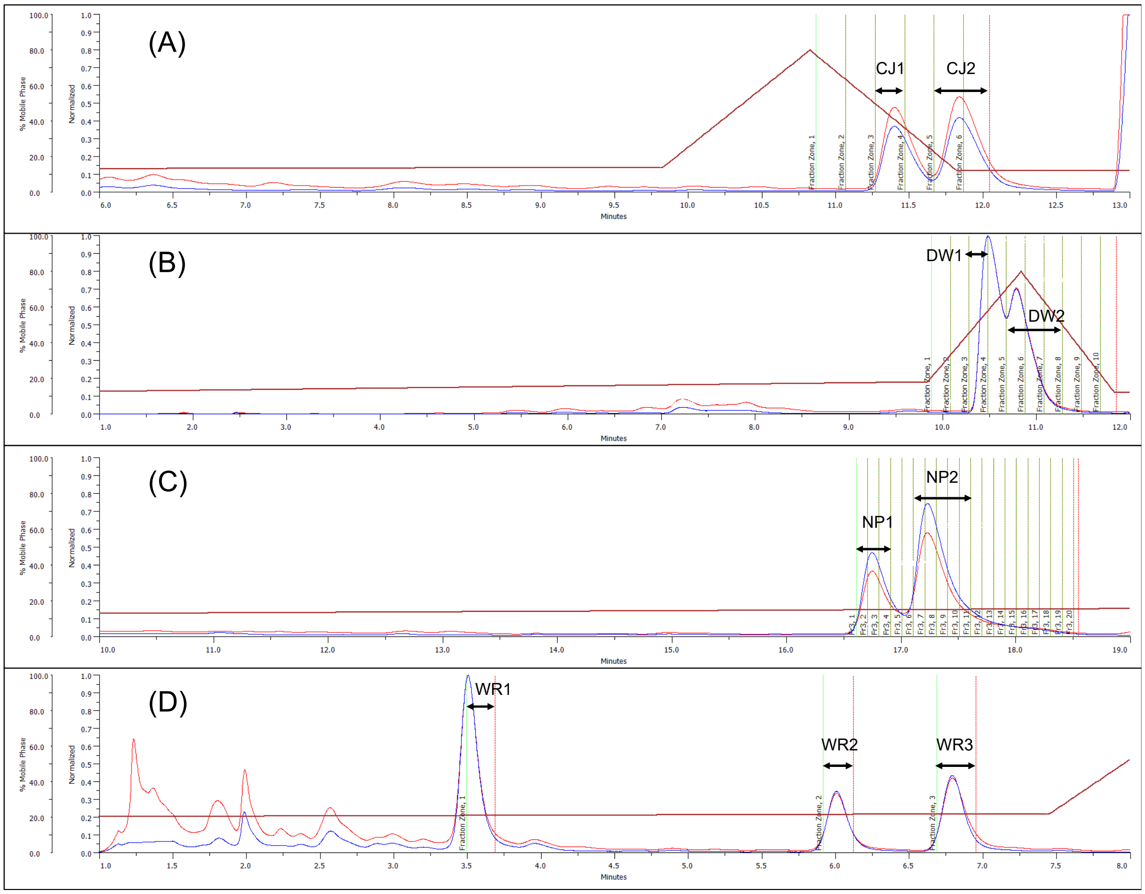

Figure S1. Preparative HPLC chromatograms for the purified compounds of soybean leaves. Preparative separation of (A) two compounds from soybean leaf (SL) of CJ variety, (B) two compounds from SL of DW variety, (C) two compounds from SL of NP variety, (D) three compounds from SL of CJ variety. Horizontal two-way arrows are indicating the collected fraction for the named peaks; red peak lines, 245 nm; blue peak lines, 365 nm; red bold lines, mobile phase B%. Elution programs (min–B%): (A) 0–12, 10–14, 11–80, 12–12, and 16–12, (B) 0–12, 10–18, 11–80, 12–12, and 16–12, (C) 0–10, 20–16, 21–80, 22–10, and 27–10, and (D) 0–20, 8–22, 9–80, 10–20, and 14–20.

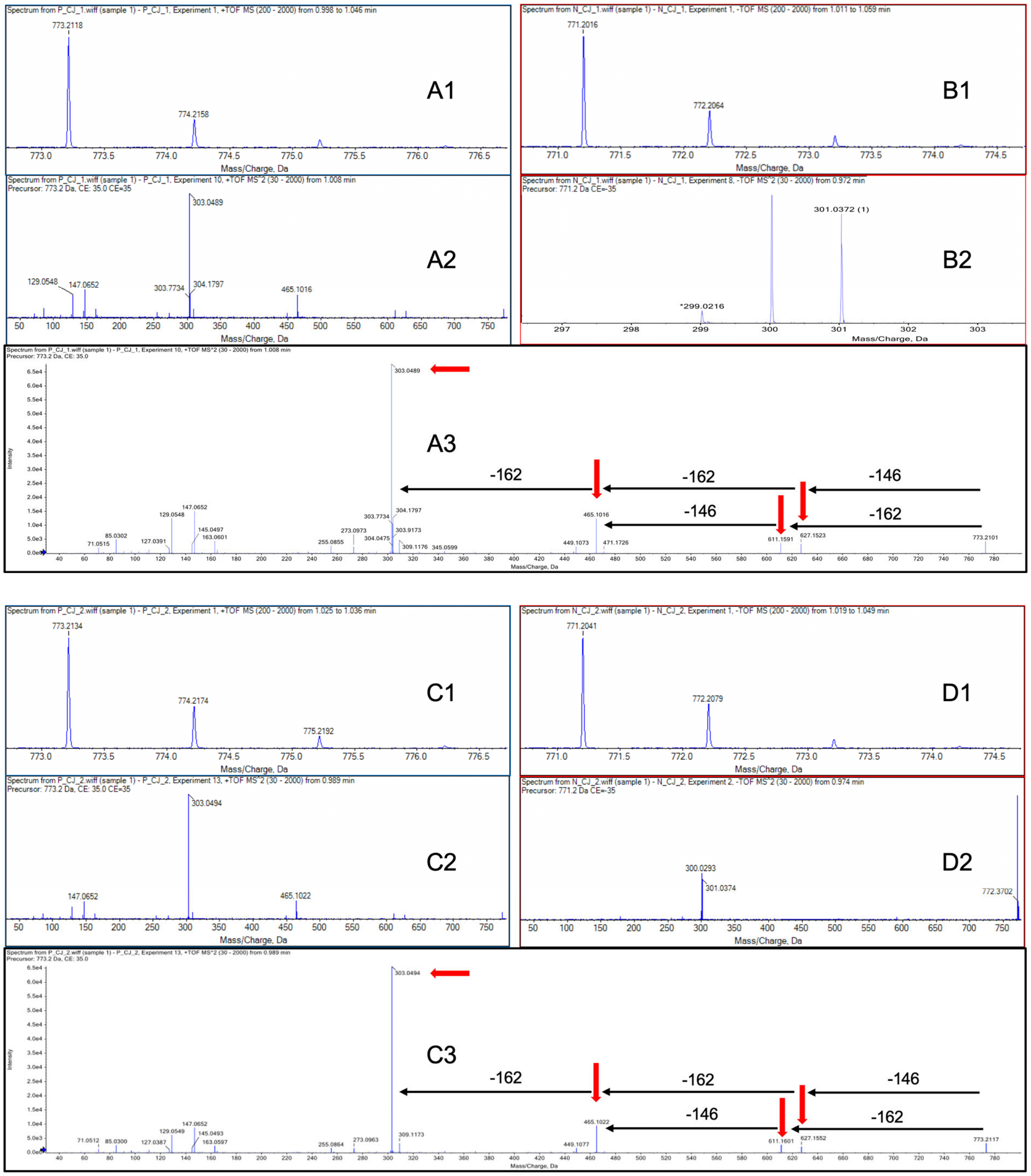

Figure S2. Mass spectra of precursors and fragments of purified compounds from CJ.

A1, precursor of CJ1 compound at positive mode; A2, fragment of CJ1 compound at positive mode;

A3, detailed fragment of CJ1 at positive mode;

B1, precursor of CJ1 compound at negative mode; B2, fragment of CJ1 compound at negative mode;

C1, precursor of CJ2 compound at positive mode; C2, fragment of CJ2 compound at positive mode;

C3, detailed fragment of CJ2 at positive mode;

D1, precursor of CJ2 compound at negative mode; and D2, fragment of CJ2 compound at negative mode.

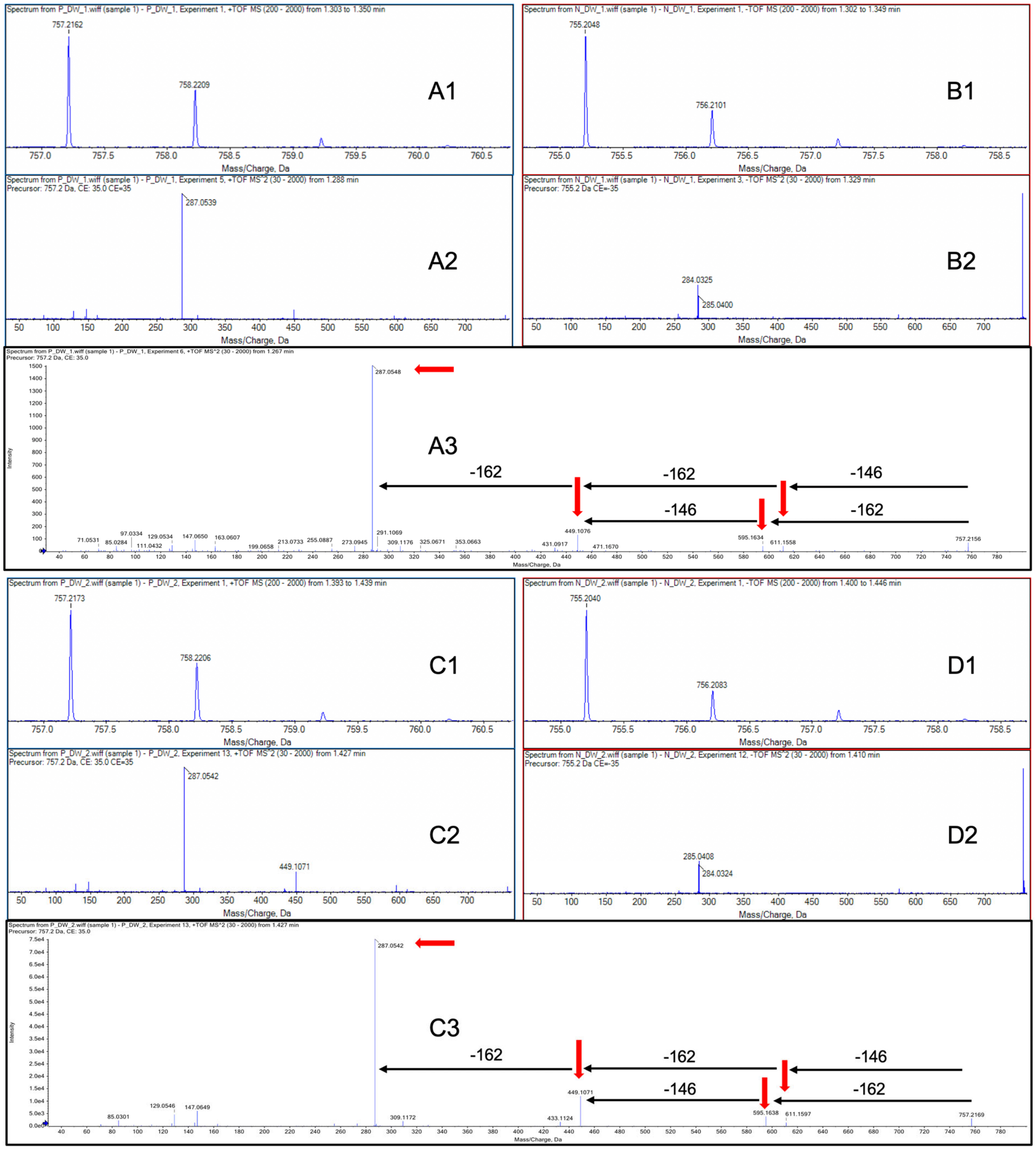

Figure S3. Mass spectra of precursors and fragments of purified compounds from DW.

A1, precursor of DW1 compound at positive mode; A2, fragment of DW1 compound at positive mode; A3, detailed fragment of DW1 at positive mode;

B1, precursor of DW1 compound at negative mode; B2, fragment of DW1 compound at negative mode;

C1, precursor of DW2 compound at positive mode; C2, fragment of DW2 compound at positive mode; C3, detailed fragment of DW2 at positive mode;

D1, precursor of DW2 compound at negative mode; and D2, fragment of DW2 compound at negative mode.

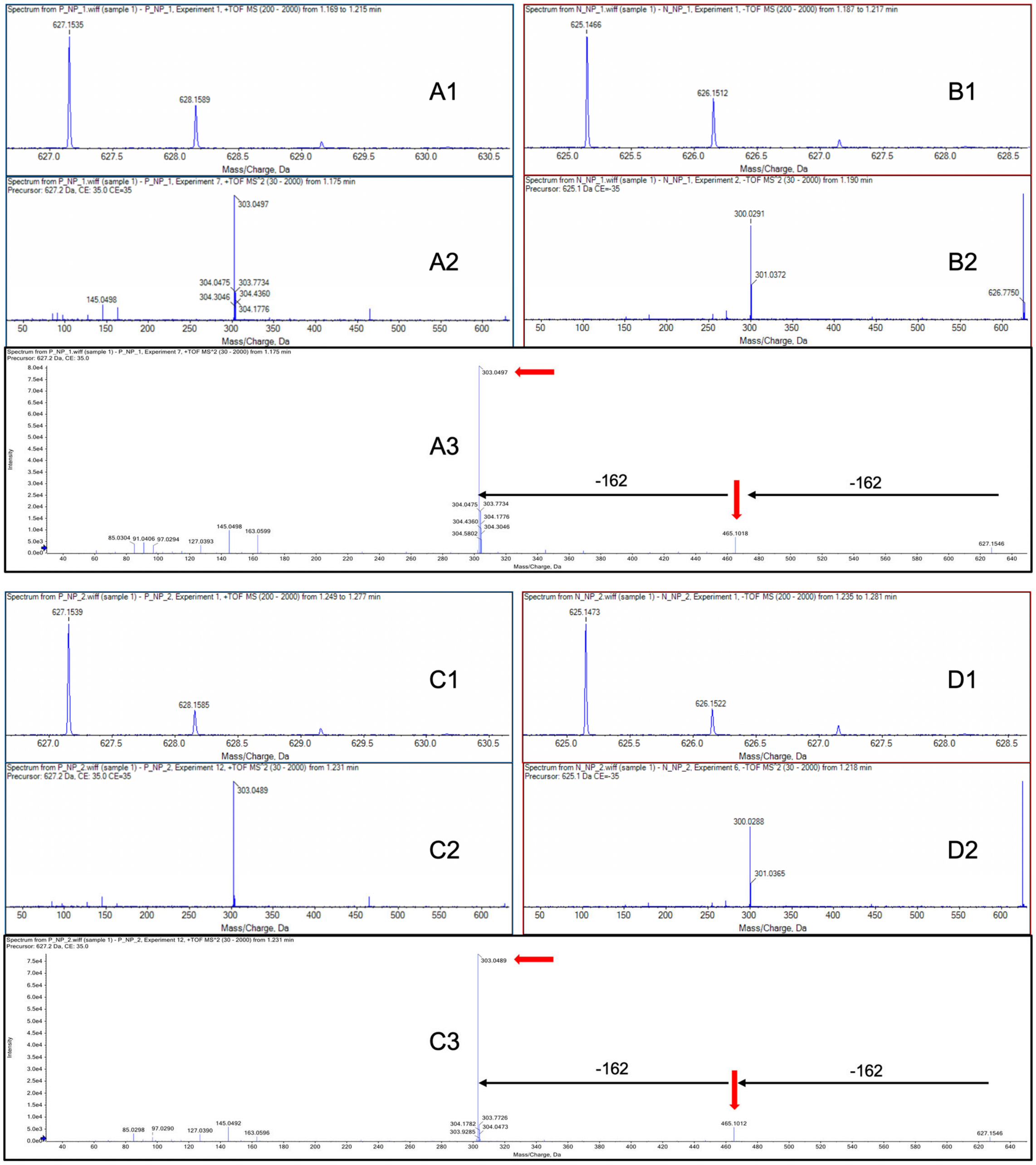

Figure S4. Mass spectra of precursors and fragments of purified compounds from NP.

A1, precursor of NP1 compound at positive mode; A2, fragment of NP1 compound at positive mode;

A3, detailed fragment of NP1 at positive mode;

B1, precursor of NP1 compound at negative mode; B2, fragment of NP1 compound at negative mode;

C1, precursor of NP2 compound at positive mode; C2, fragment of NP2 compound at positive mode;

C3, detailed fragment of NP2 at positive mode;

D1, precursor of NP2 compound at negative mode; and D2, fragment of NP2 compound at negative mode.

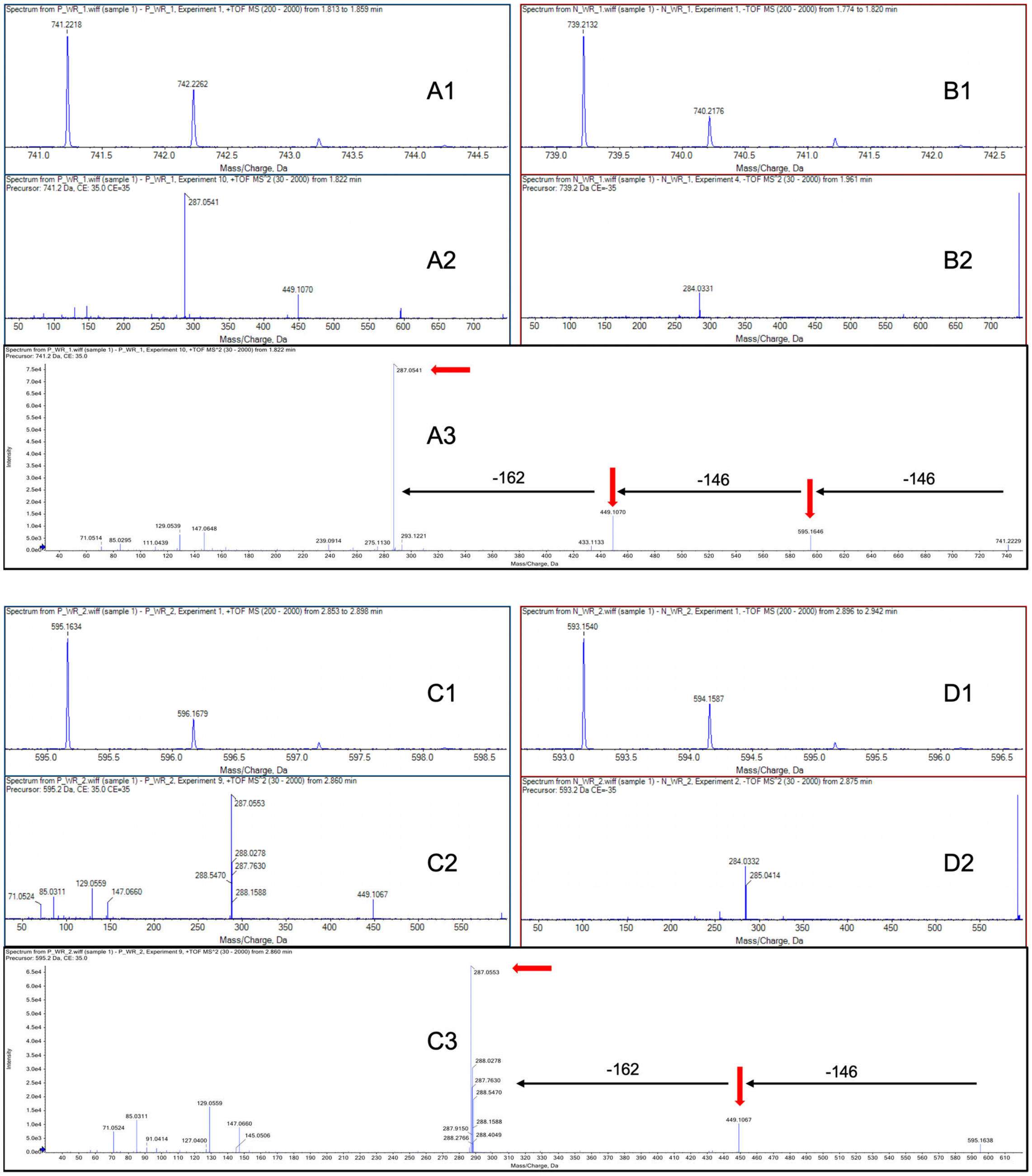
A1
741.2218
742.2262
B1
739.2132
740.2176
A2
287.0541
449.1070
B2
284.0331
A3
287.0541
-162
-146
-146
449.1070
595.1646
741.2229
Mass/Charge, Da
C1
595.1634
596.1679
D1
593.1540
594.1587
C2
287.0553
449.1067
D2
284.0332
285.0414
C3
287.0553
-162
-146
449.1067
595.1638

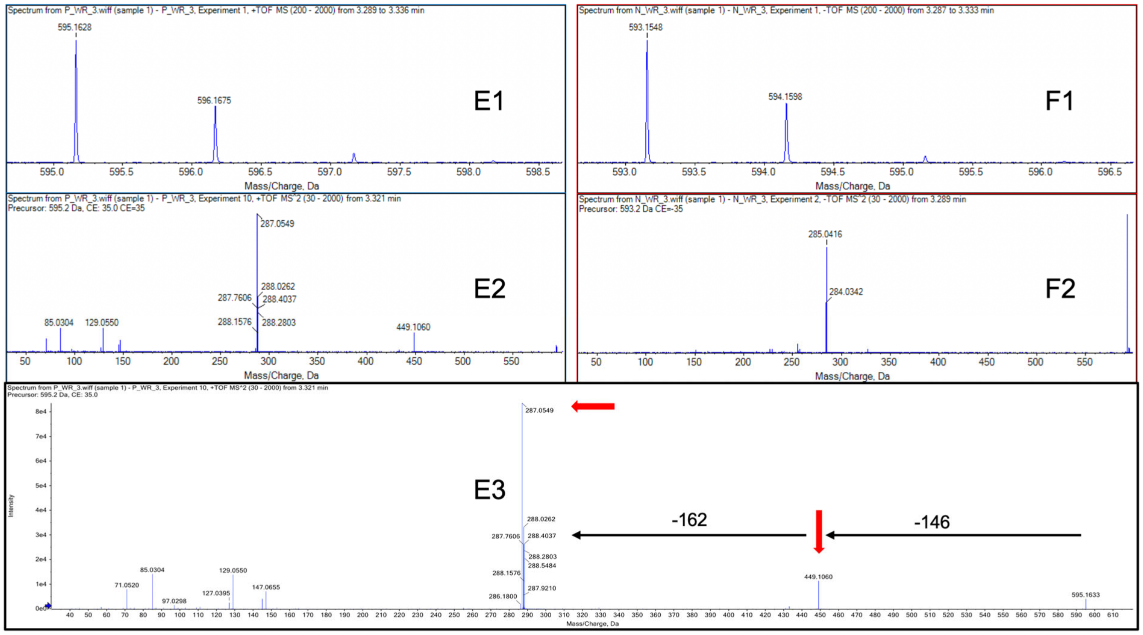

Figure S5. Mass spectra of precursors and fragments of purified compounds from WR.

A1, precursor of WR1 compound at positive mode; A2, fragment of WR1 compound at positive mode;

A3, detailed fragment of WR1 at positive mode;

B1, precursor of WR1 compound at negative mode; B2, fragment of WR1 compound at negative mode;

C1, precursor of WR2 compound at positive mode; C2, fragment of WR2 compound at positive mode;

C3, detailed fragment of WR2 at positive mode;

D1, precursor of WR2 compound at negative mode; D2, fragment of WR2 compound at negative mode;

E1, precursor of WR3 compound at positive mode; E2, fragment of WR3 compound at positive mode;

E3, detailed fragment of WR3 at positive mode;

F1, precursor of WR3 compound at negative mode; and F2, fragment of WR3 compound at negative mode

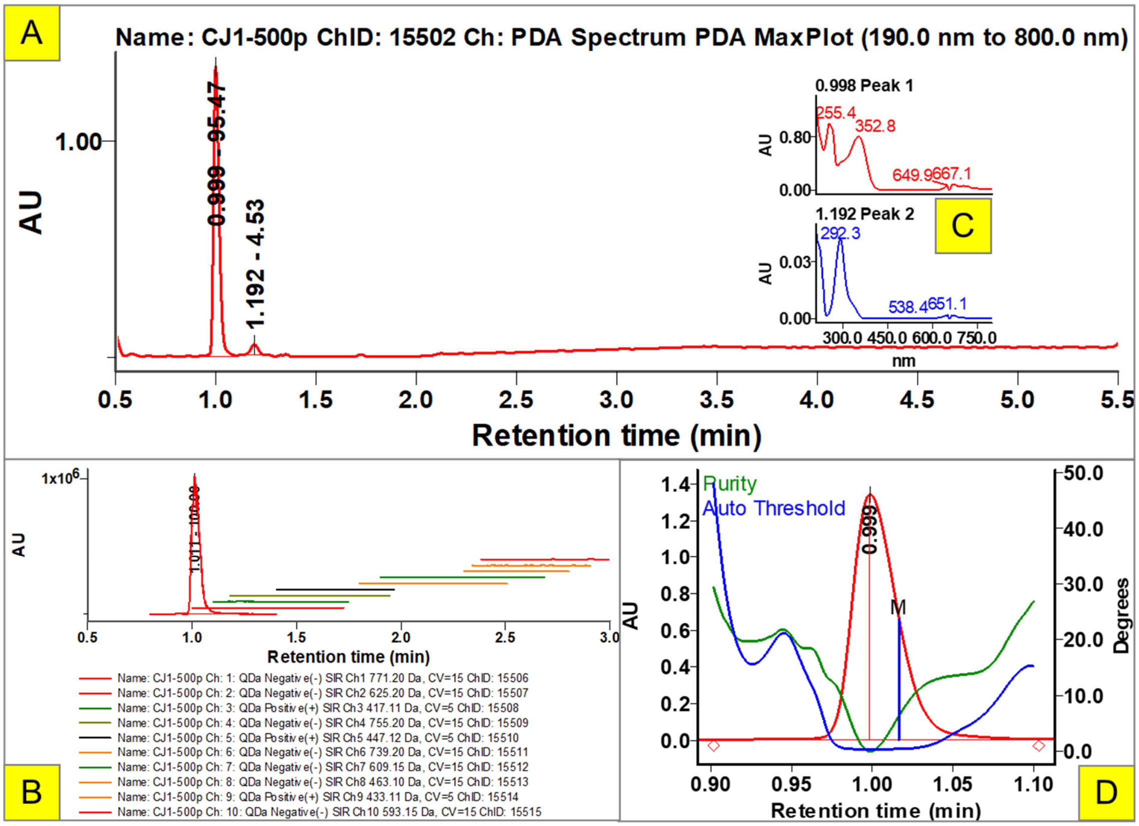

Figure S6. Spectral purity calculation for the purified compounds CJ1.

(A), PDA spectrum of the purified compound; (B), mass spectrum of the purified compound; (C), PDA spectrum of the peaks in the (A); (D), purity of the major peak analysed by Empower 3 software.

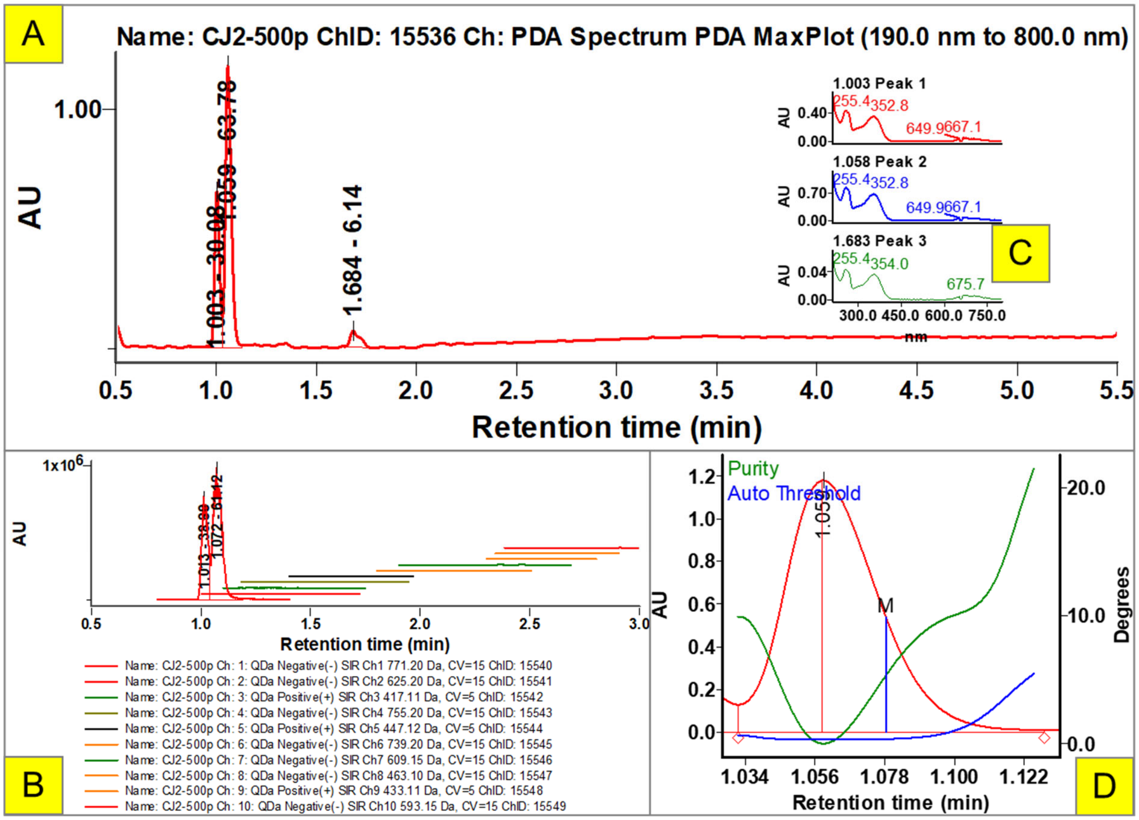

Figure S7. Spectral purity calculation for the purified compounds CJ2.

(A), PDA spectrum of the purified compound; (B), mass spectrum of the purified compound; (C), PDA spectrum of the peaks in the (A); (D), purity of the major peak analysed by Empower 3 software.

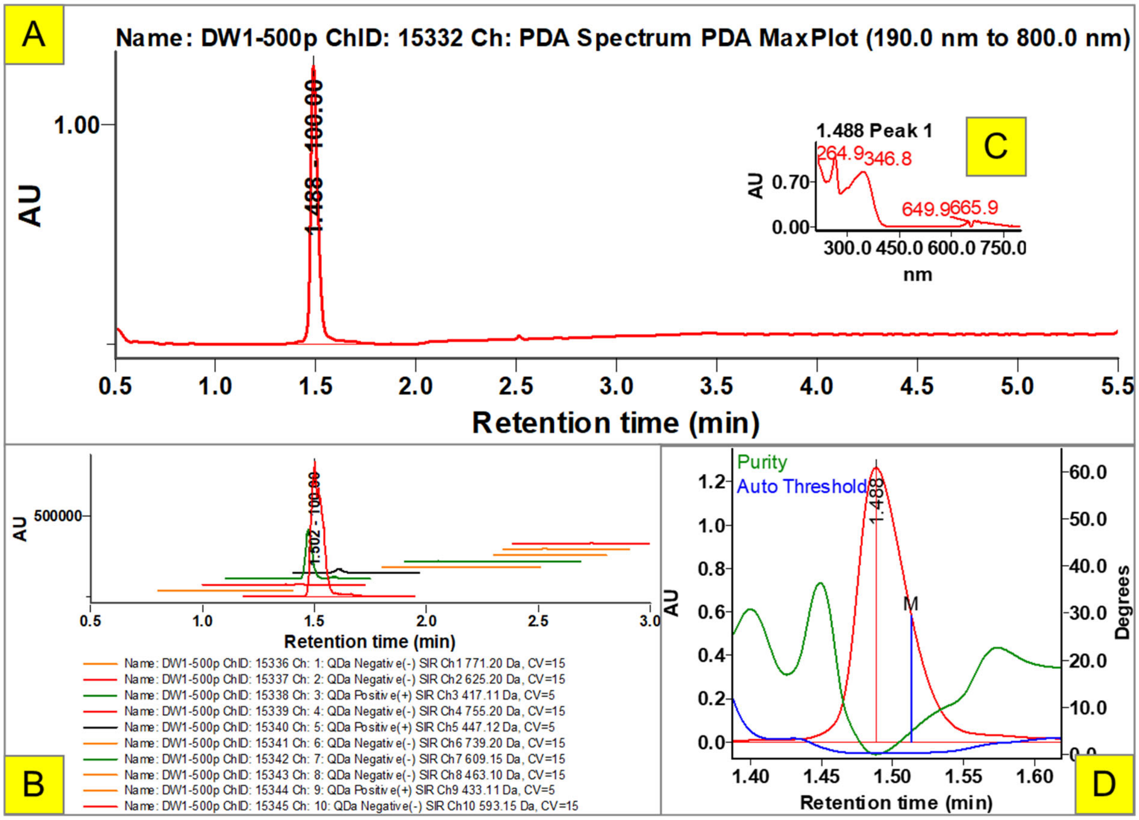

Figure S8. Spectral purity calculation for the purified compounds DW1.

(A), PDA spectrum of the purified compound; (B), mass spectrum of the purified compound; (C), PDA spectrum of the peaks in the (A); (D), purity of the major peak analysed by Empower 3 software.

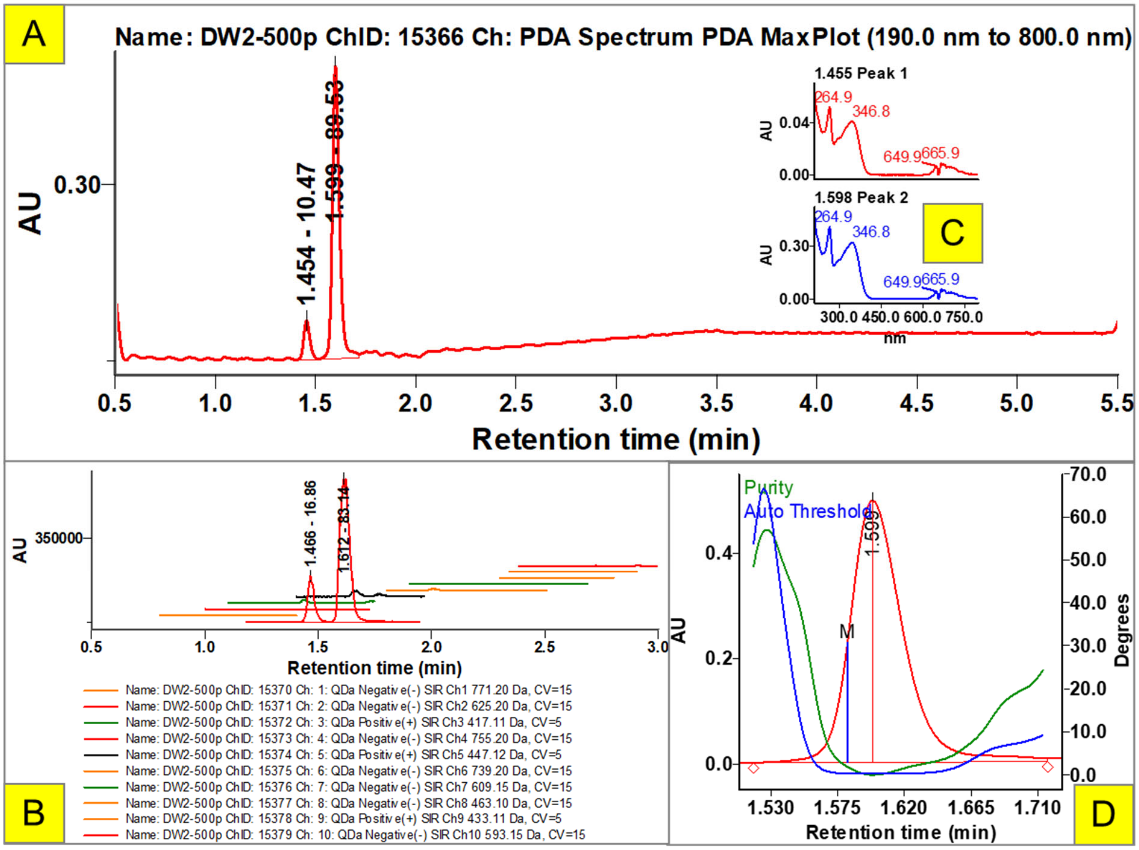

Figure S9. Spectral purity calculation for the purified compounds DW2.

(A), PDA spectrum of the purified compound; (B), mass spectrum of the purified compound; (C), PDA spectrum of the peaks in the (A); (D), purity of the major peak analysed by Empower 3 software.

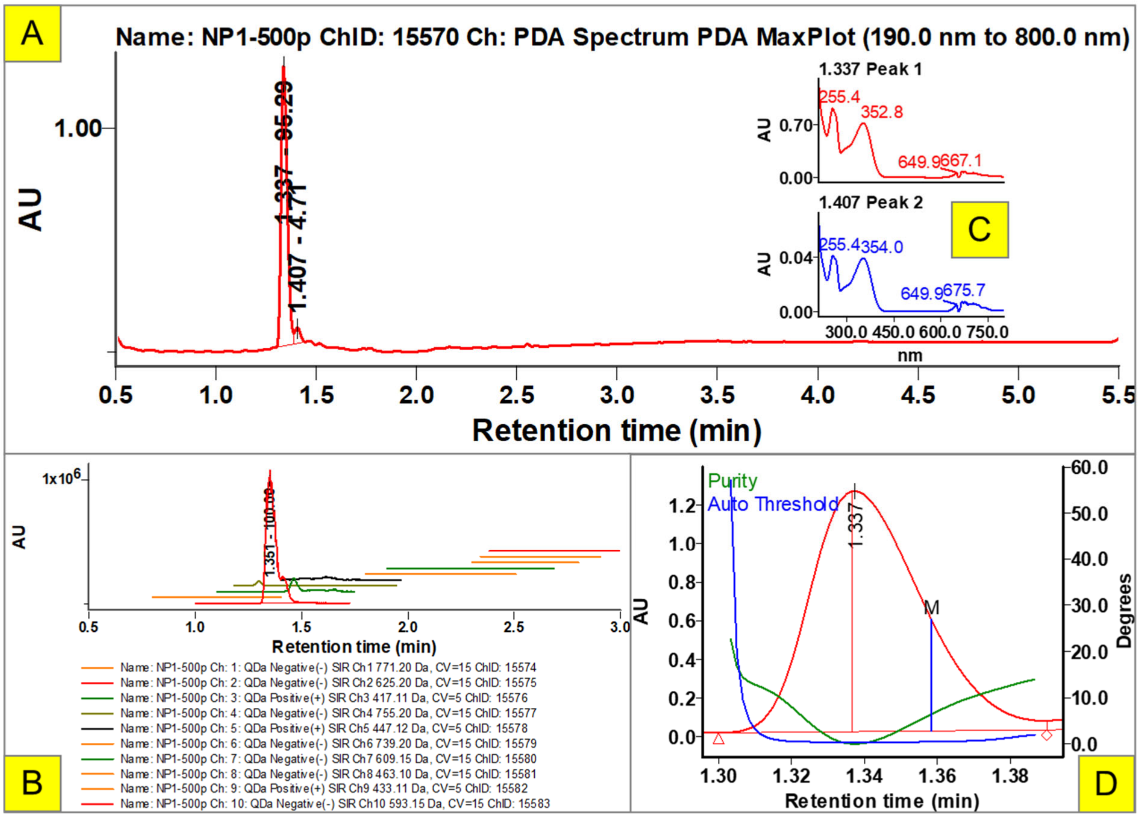

Figure S10. Spectral purity calculation for the purified compounds NP1.

(A), PDA spectrum of the purified compound; (B), mass spectrum of the purified compound; (C), PDA spectrum of the peaks in the (A); (D), purity of the major peak analysed by Empower 3 software.

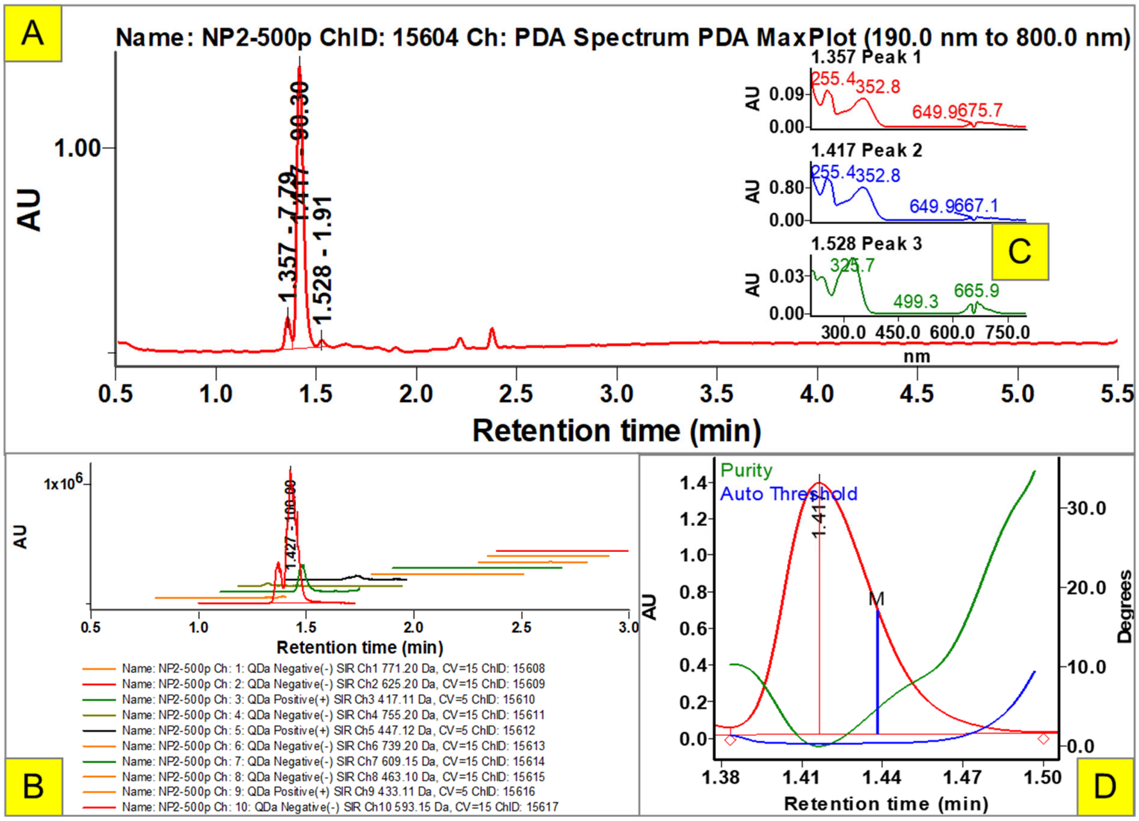

Figure S11. Spectral purity calculation for the purified compounds NP2.

(A), PDA spectrum of the purified compound; (B), mass spectrum of the purified compound; (C), PDA spectrum of the peaks in the (A); (D), purity of the major peak analysed by Empower 3 software.

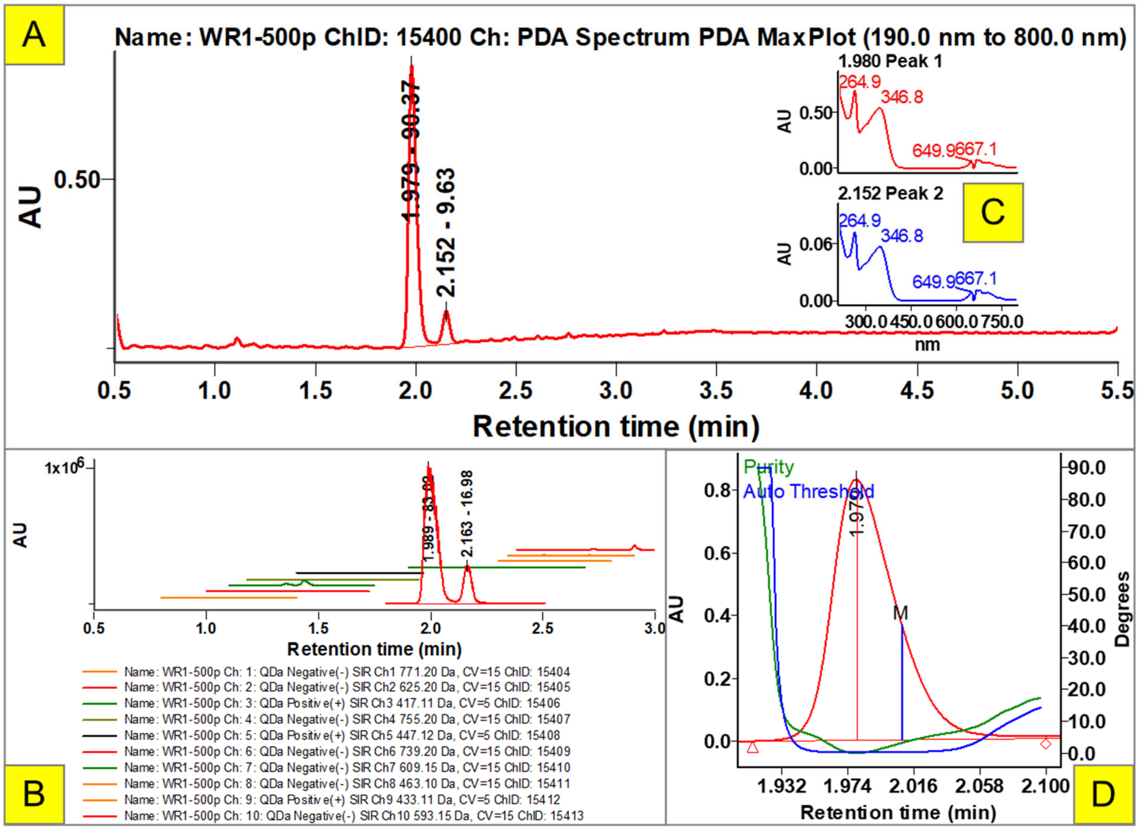

Figure S12. Spectral purity calculation for the purified compounds WR1.

(A), PDA spectrum of the purified compound; (B), mass spectrum of the purified compound; (C), PDA spectrum of the peaks in the (A); (D), purity of the major peak analysed by Empower 3 software.

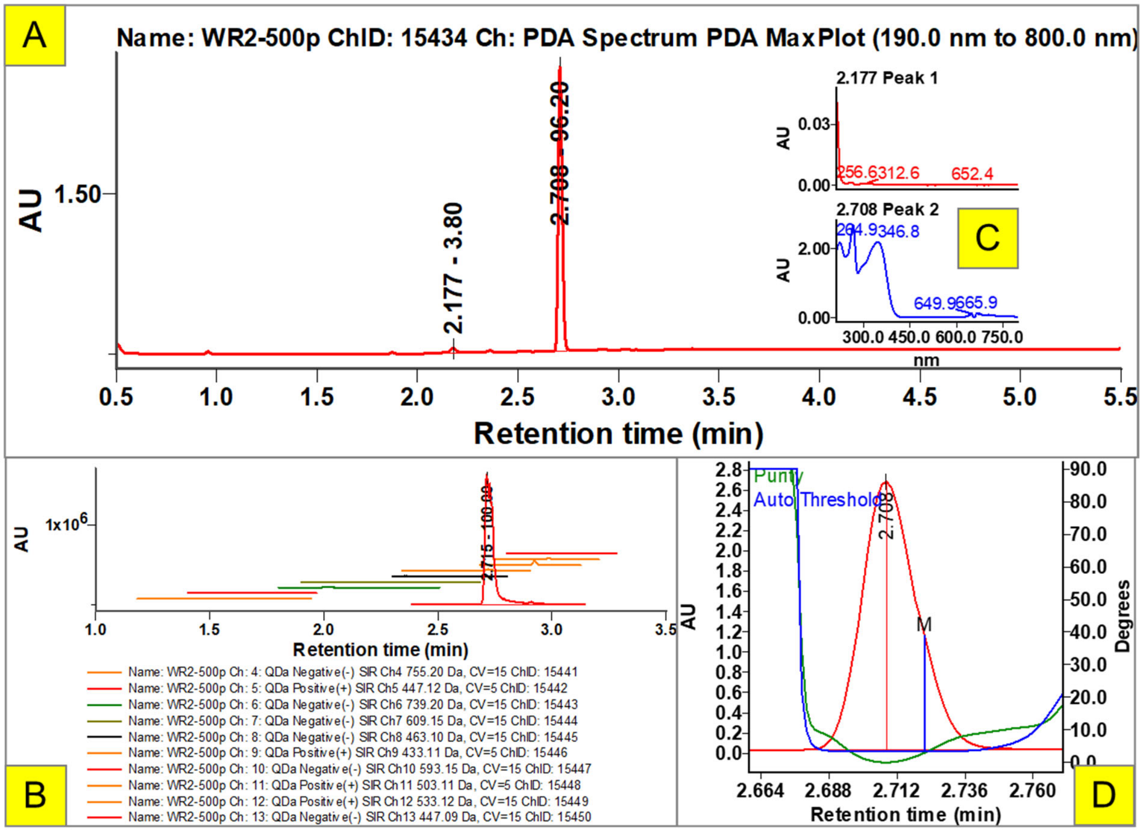

Figure S13. Spectral purity calculation for the purified compounds WR2.

(A), PDA spectrum of the purified compound; (B), mass spectrum of the purified compound; (C), PDA spectrum of the peaks in the (A); (D), purity of the major peak analysed by Empower 3 software.

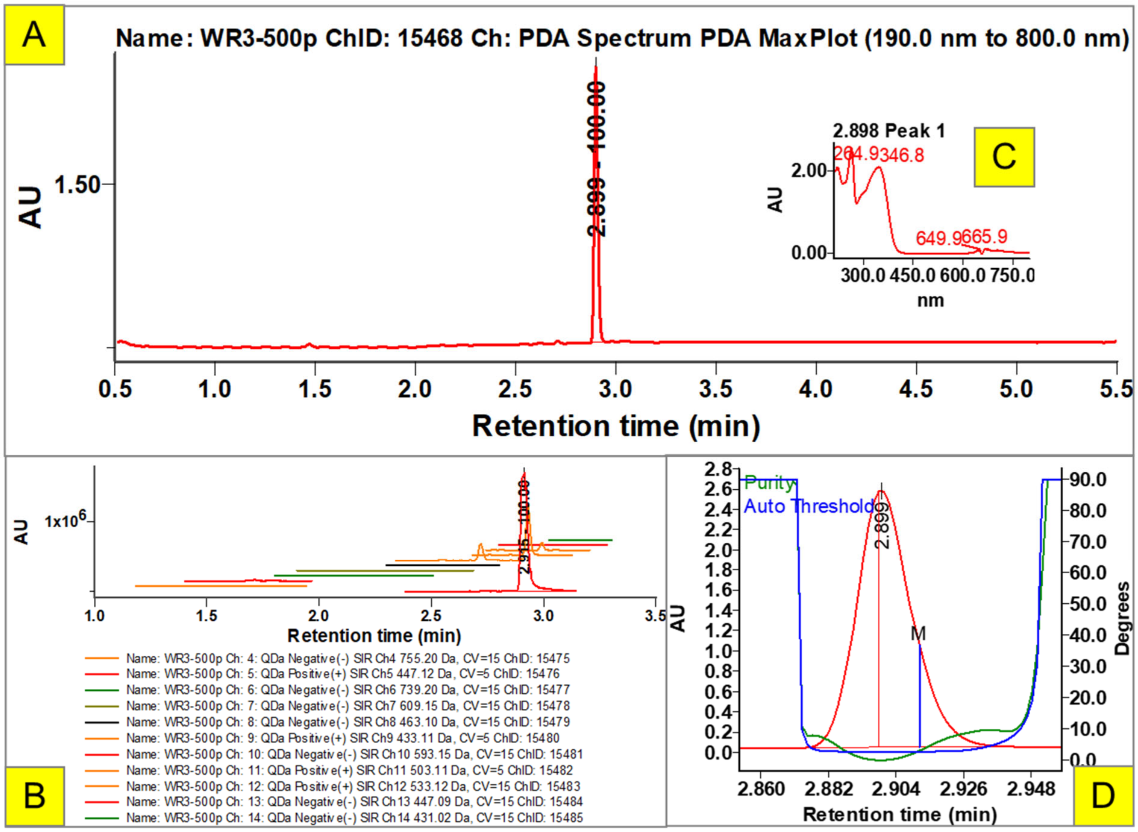

Figure S14. Spectral purity calculation for the purified compounds WR3.

(A), PDA spectrum of the purified compound; (B), mass spectrum of the purified compound; (C), PDA spectrum of the peaks in the (A); (D), purity of the major peak analysed by Empower 3 software.

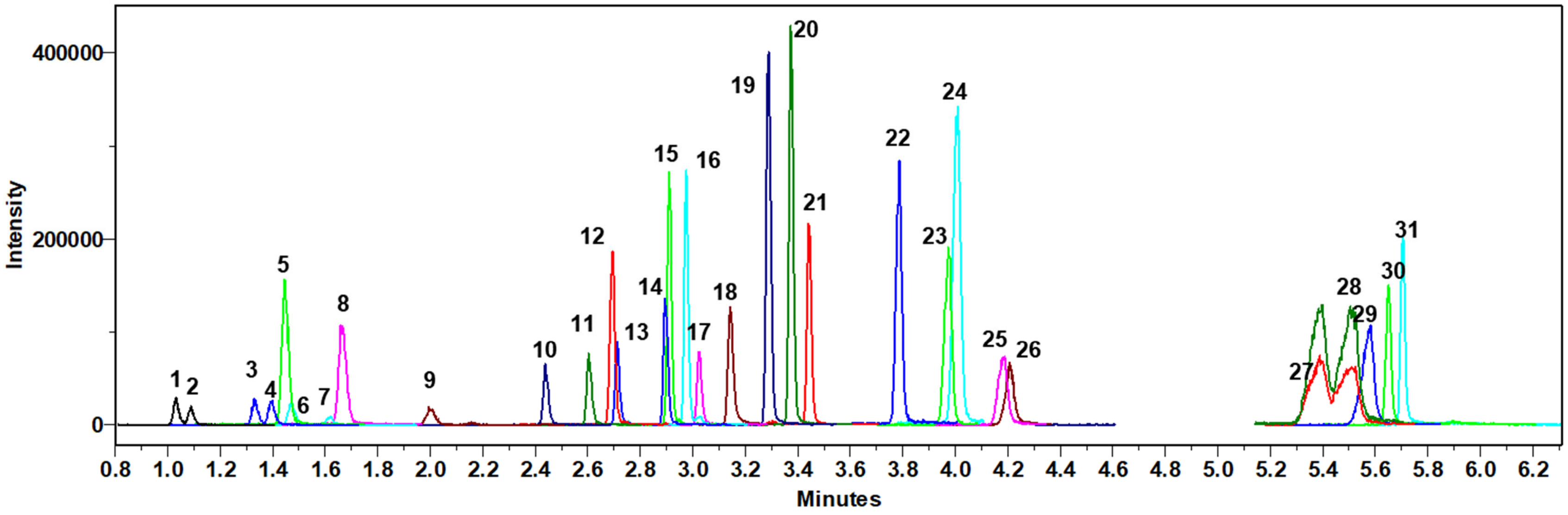

Figure S15. Overlaid single ion recording mass chromatogram of 31 compounds.

1, quercetin 3-*O*-tri-glycoside A; **2**, quercetin 3-*O*-tri-glycoside B; **3**, quercetin 3-*O*-di-glycoside A; **4**, quercetin 3-*O*-di-glycoside B; **5**, daidzin; **6**, kaempferol 3-*O*-tri-glycoside A; **7**, kaempferol 3-*O*-tri-glycoside B; **8**, glycitin; **9**, kaempferol 3-*O*-tri-glycoside C; **10**, rutin; **11**, isoquercitrin; **12**, genistin; **13**, kaempferol 3-*O*-di-glycoside A; **14**, kaempferol 3-*O*-di-glycoside B; **15**, malonyl daidzin; **16**, malonyl glycitin; **17**, astragalin; **18**, apigenin 7-*O*-glucoside; **19**, acetyl daidzin; **20**, acetyl glycitin; **21**, malonyl genistin; **22**, daidzein; **23**, acetyl genistin; **24**, glycitein; **25**, luteolin; **26**, quercetin; **27**, genistein; **28**, apigenin; **29**, coumestrol ; **30**, kaempferol; and **31**, isorhamnetin. Refer to details of the compounds in Table S1.

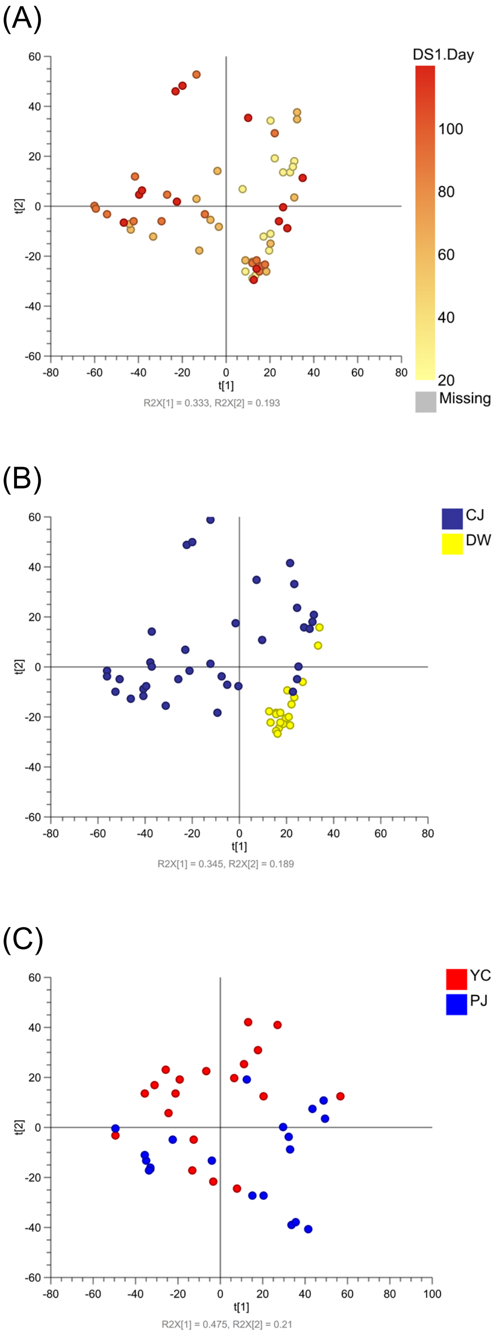

Figure S16. Multivariate analysis of SL metabolites.

(A) PCA-XY score plot for cultivation day, and PCA-X score plots for (B) variety of SB and (C) cultivation sites.

(A)

Neural

Validation Column: Validation0.6

Model NTanH(3)NBoost(6)

Training — Day

Measures	Value
Generalized RSquare	0.9999255
Entropy RSquare	0.9996209
RMSE	0.0011122
Mean Abs Dev	0.0005087
Misclassification Rate	0
-LogLikelihood	0.0178279
Sum Freq	35

Confusion Matrix

Actual Day	Predicted Count 30	60	90	120
30	5	0	0	0
60	0	9	0	0
90	0	0	9	0
120	0	0	0	12

Confusion Rates

Actual Day	Predicted Rate 30	60	90	120
30	1.000	0.000	0.000	0.000
60	0.000	1.000	0.000	0.000
90	0.000	0.000	1.000	0.000
120	0.000	0.000	0.000	1.000

Validation — Day

Measures	Value
Generalized RSquare	0.9864539
Entropy RSquare	0.9410184
RMSE	0.115642
Mean Abs Dev	0.0674734
Misclassification Rate	0
-LogLikelihood	0.9102448
Sum Freq	12

Confusion Matrix

Actual Day	Predicted Count 30	60	90	120
30	3	0	0	0
60	0	4	0	0
90	0	0	4	0
120	0	0	0	1

Confusion Rates

Actual Day	Predicted Rate 30	60	90	120
30	1.000	0.000	0.000	0.000
60	0.000	1.000	0.000	0.000
90	0.000	0.000	1.000	0.000
120	0.000	0.000	0.000	1.000

Test — Day

Measures	Value
Generalized RSquare	0.9596596
Entropy RSquare	0.8630507
RMSE	0.2268725
Mean Abs Dev	0.1100007
Misclassification Rate	0.0909091
-LogLikelihood	1.7603052
Sum Freq	11

Confusion Matrix

Actual Day	Predicted Count 30	60	90	120
30	5	1	0	0
60	0	2	0	0
90	0	0	2	0
120	0	0	0	1

Confusion Rates

Actual Day	Predicted Rate 30	60	90	120
30	0.833	0.167	0.000	0.000
60	0.000	1.000	0.000	0.000
90	0.000	0.000	1.000	0.000
120	0.000	0.000	0.000	1.000

(B)

Neural

Validation Column: Validation0.6

Model NTanH(3)NBoost(10)

Training — Variety

Measures	Value
Generalized RSquare	0.9999956
Entropy RSquare	0.9999912
RMSE	1.4853e-5
Mean Abs Dev	5.4621e-6
Misclassification Rate	0
-LogLikelihood	0.0001912
Sum Freq	35

Confusion Matrix

Actual Variety	Predicted Count CJ	DW
CJ	24	0
DW	0	11

Confusion Rates

Actual Variety	Predicted Rate CJ	DW
CJ	1.000	0.000
DW	0.000	1.000

Validation — Variety

Measures	Value
Generalized RSquare	0.999931
Entropy RSquare	0.9998507
RMSE	0.0002302
Mean Abs Dev	0.0001034
Misclassification Rate	0
-LogLikelihood	0.0012416
Sum Freq	12

Confusion Matrix

Actual Variety	Predicted Count CJ	DW
CJ	6	0
DW	0	6

Confusion Rates

Actual Variety	Predicted Rate CJ	DW
CJ	1.000	0.000
DW	0.000	1.000

Test — Variety

Measures	Value
Generalized RSquare	0.9996669
Entropy RSquare	0.9994448
RMSE	0.000478
Mean Abs Dev	0.0002631
Misclassification Rate	0
-LogLikelihood	0.0028959
Sum Freq	11

Confusion Matrix

Actual Variety	Predicted Count CJ	DW
CJ	9	0
DW	0	2

Confusion Rates

Actual Variety	Predicted Rate CJ	DW
CJ	1.000	0.000
DW	0.000	1.000

(C)

Neural

Validation Column: Validation0.6

Model NTanH(3)NBoost(2)

Training — City

Measures	Value
Generalized RSquare	0.9999654
Entropy RSquare	0.999926
RMSE	0.0001493
Mean Abs Dev	5.0547e-5
Misclassification Rate	0
-LogLikelihood	0.0017695
Sum Freq	35

Confusion Matrix

Actual City	Predicted Count PJ	YC
PJ	20	0
YC	0	15

Confusion Rates

Actual City	Predicted Rate PJ	YC
PJ	1.000	0.000
YC	0.000	1.000

Validation — City

Measures	Value
Generalized RSquare	0.9981204
Entropy RSquare	0.9974634
RMSE	0.0014243
Mean Abs Dev	0.0007266
Misclassification Rate	0
-LogLikelihood	0.008731
Sum Freq	12

Confusion Matrix

Actual City	Predicted Count PJ	YC
PJ	11	0
YC	0	1

Confusion Rates

Actual City	Predicted Rate PJ	YC
PJ	1.000	0.000
YC	0.000	1.000

Test — City

Measures	Value
Generalized RSquare	0.9911419
Entropy RSquare	0.9833221
RMSE	0.0226218
Mean Abs Dev	0.0095058
Misclassification Rate	0
-LogLikelihood	0.1074969
Sum Freq	11

Confusion Matrix

Actual City	Predicted Count PJ	YC
PJ	8	0
YC	0	3

Confusion Rates

Actual City	Predicted Rate PJ	YC
PJ	1.000	0.000
YC	0.000	1.000

Figure S17. Prediction results of Neural model of machine learning.

Discriminated by (A) day, (B) variety, and (C) city. All predictions were performed with same dataset comprised of randomly allocated training, validation, and test set.

Script S1. Python code for prediction of the discriminative classes.

```
""" NOTE: "jmp_score.py" file must be in the same directory to perform the
predictions"""

""" import generated python codes for prediction from JMP """
import neural_day
import neural_variety
import neural_city
import BF_day
import BT_variety
import BT_city

""" import library for data analysis """
import pandas as pd

""" read test dataset """
df = pd.read_excel('test_dataset.xlsx')

""" apply the prediction code to the test dataset and append the results"""
df['pred_neural_day'] = df[neural_day.getInputMetadata()].apply(lambda s:
neural_day.score(s, {}), axis=1)
df['pred_neural_variety'] =
df[neural_variety.getInputMetadata()].apply(lambda s:
neural_variety.score(s, {}), axis=1)
df['pred_neural_city'] = df[neural_city.getInputMetadata()].apply(lambda s:
neural_city.score(s, {}), axis=1)
df['pred_BF_day'] = df[BF_day.getInputMetadata()].apply(lambda s:
BF_day.score(s, {}), axis=1)
df['pred_BT_variety'] = df[BT_variety.getInputMetadata()].apply(lambda s:
BT_variety.score(s, {}), axis=1)
df['pred_BT_city'] = df[BT_city.getInputMetadata()].apply(lambda s:
BT_city.score(s, {}), axis=1)


""" print the predictions with the originals"""
prd=
df[['Day','pred_neural_day','pred_BF_day','Variety','pred_neural_variety','
pred_BT_variety', 'City','pred_neural_city','pred_BT_city']]
prd
```

*Designated file: ML_prediction.py

To obtain the designated results, following files must be in the same directory; neural_day.py, neural_variety.py, neural_city.py, BF_day.py, BT_variety.py, BT_city.py, jmp_score.py, and test_dataset.xlsx. Refer to the printed results in Table S9. The code can be executed in Python (Python Software Foundation; www.python.org) environments.
